# LDS1-produced oxylipins are negative regulators of growth, conidiation and fumonisin synthesis in the fungal maize pathogen *Fusarium verticillioides*

**DOI:** 10.3389/fmicb.2014.00669

**Published:** 2014-12-11

**Authors:** Valeria Scala, Paola Giorni, Martina Cirlini, Matteo Ludovici, Ivan Visentin, Francesca Cardinale, Anna A. Fabbri, Corrado Fanelli, Massimo Reverberi, Paola Battilani, Gianni Galaverna, Chiara Dall'Asta

**Affiliations:** ^1^Department of Environmental Biology, University of Rome “Sapienza”Rome, Italy; ^2^Istituto di Entomologia e Patologia Vegetale, Università Cattolica del Sacro CuorePiacenza, Italy; ^3^Food Chemistry and Natural Substances Unit, Department of Organic and Industrial Chemistry, University of ParmaParma, Italy; ^4^Department of Agricultural, Food and Forestry Science, University of TurinTorino, Italy

**Keywords:** *Fusarium verticillioides*, *Zea mays*, lipidomic, genome sequencing, chromatin immunoprecipitation

## Abstract

Oxylipins are fatty acid-derived signaling compounds produced by all eukaryotes so far investigated; in mycotoxigenic fungi, they modulate toxin production and interactions with the host plants. Among the many enzymes responsible for oxylipin generation, Linoleate Diol Synthase 1 (LDS1) produces mainly 8-hydroperoxyoctadecenoic acid and subsequently different di-hydroxyoctadecenoic acids. In this study, we inactivated a copy of the putative *LDS1* ortholog (acc. N. FVEG_09294.3) of *Fusarium verticillioides*, with the aim to investigate its influence on the oxylipin profile of the fungus, on its development, secondary metabolism and virulence. LC-MS/MS oxylipin profiling carried out on the selected mutant strain revealed significant quali-quantitative differences for several oxylipins when compared to the WT strain. The Fv*lds1*-deleted mutant grew better, produced more conidia, synthesized more fumonisins and infected maize cobs faster than the WT strain. We hypothesize that oxylipins may act as regulators of gene expression in the toxigenic plant pathogen *F. verticillioides*, in turn causing notable changes in its phenotype. These changes could relate to the ability of oxylipins to re-shape the transcriptional profile of *F. verticillioides* by inducing chromatin modifications and exerting a direct control on the transcription of secondary metabolism in fungi.

## Introduction

*Fusarium verticillioides* causes the ear and stalk rot of maize (*Zea mays*) by entering through roots, stalks and ears at different growth stages. Tissue invasion is often asymptomatic even in the presence of massive growth inside the kernels (Estrada et al., 2012), where *F. verticillioides* produces fumonisins. This is a family of mycotoxins that, especially in their B series (FB), are hazardous for human and animal health, while apparently not directly related to fungal virulence and aggressiveness. In particular, fumonisin B_1_ (FB_1_) has been included in the 2B class by the International Agency for Research on Cancer because of its possible carcinogenic effect in humans. Moreover, the European Union has enforced the legislation to fix a threshold of FB_1_ + FB_2_ content in raw maize and derived products intended for human consumption (EU Commission Regulation No. 1126/2007); recommendations have also been given for animal feeding (EU Commission Regulation No. 576/2006).

Fatty acids (FAs) and FA-metabolites are major structural and metabolic constituents of the cell, functioning also as modulators of signal transduction pathways or transcription factors induced by several stimuli (Duplus et al., 2000). For instance, the enzymatic and non-enzymatic peroxidation of FAs is one of the processes switched on during early plant defense signaling related to pathogen perception (Shea and Del Poeta, 2006; Walley et al., 2013). The products of FA peroxidation, named oxylipins, constitute a large family of oxidized FAs, and their by-products are present in almost every living kingdom (Koo and Howe, 2009; Brodhun and Feussner, 2011). The non-enzymatic pathway of oxylipin synthesis derives from spontaneous formation of reactive oxygen species (ROS) such as hydroxyl radical, which may initiate lipid peroxidation (Brodhun and Feussner, 2011). Besides this route, oxylipins are mainly produced through three enzyme-dependent pathways, the ones initiated by lipoxygenases (LOX) or dioxygenases, and pathways involving cytochromes (Brodhun and Feussner, 2011). Oxylipins possess pivotal functions as signal molecules (Christensen and Kolomiets, 2011).

In fungi, the first described oxylipins (Precocious sexual inducers, Psi-factors) owe the name to their involvement in the regulation of the sexual and asexual phases in *Aspergillus* (Tsitsigiannis et al., 2005; Brown et al., 2008). Psi factors are also involved in host-pathogen communication and promote fungal infection of plants (Tsitsigiannis and Keller, 2006; Brodhun and Feussner, 2011; Christensen and Kolomiets, 2011). The enzymes responsible for synthesizing the Psi-factors were initially identified in *Aspergillus nidulans* as orthologs of the 7,8-linoleate diol synthase (7,8-LDS) from *Gaeumannomyces graminis*, and were named Psi factor-producing oxygenases (Ppo) (Tsitsigiannis et al., 2005; Garscha et al., 2007). Over recent years, 7,8-LDS has been considered functionally and structurally similar to the mammalian prostaglandin H2-synthase. 7,8-LDS-encoding genes have been detected in several pathogenic and non-pathogenic fungi such as: *Cercospora zeae-maydis* (Shim and Dunkle, 2002), *Magnaporthe grisea* (Cristea et al., 2003), *Magnaporthe oryzae* (Jerneren et al., 2010), *Ustilago maydis* (Huber et al., 2002), and *A. nidulans*. In the latter fungus, three different genes with significant similarity to the gene encoding 7,8-LDS were identified (*PpoA*, *PpoB*, and *PpoC*) (Tsitsigiannis et al., 2005) and a fourth gene (*PpoD*) was characterized in *Aspergillus flavus* (Brown et al., 2009). Information on LDS enzymatic activity is scanty: it is known that *PpoA* is responsible for the formation of 5*S*,8*R*-dihydroxyoctadecenoic acid (diHODE), whilst *PpoC* could code for a 10R-dioxygenase, producing 10-hydroperoxyoctadecenoic acid (HPODE) (Garscha et al., 2007; Brodhun et al., 2009).

Plant and fungal lipids and their oxidized derivatives may drive the fate (compatible/incompatible) of plant-pathogen interactions and mycotoxin production (Tsitsigiannis and Keller, 2007; Christensen et al., 2014; Scarpari et al., 2014). During its interaction with the host, *F. verticillioides* induces the expression of the maize LOX, Zm*LOX3* as well as of its close homolog Zm*LOX2* (Gao et al., 2007). Infections by *Aspergillus* spp. increase 9*S*-HPODE levels in peanut seeds while suppressing 13*S*-HPODE synthesis (Brodhagen et al., 2008). Mycotoxigenic fungi may hijack the host 9-LOX pathway that, in turn, induces mycotoxin biosynthesis; thus, some 9-oxylipins function as susceptibility factors in the producing host plants (Gao et al., 2007; Tsitsigiannis and Keller, 2007; Brodhagen et al., 2008; Christensen and Kolomiets, 2011; Scarpari et al., 2014). Coherently, the pathogen-derived oxylipins may induce the expression of the host *LOX* genes. Brodhagen et al. (2008) indeed showed that whilst wild-type (WT) *A. nidulans* triggers the expression of a peanut *LOX* gene, its oxylipin-deficient *ppo* mutants fail to do so. Thus, an additional function for the oxylipins produced by pathogenic fungi might be in manipulating the expression of the host genes involved in lipid and oxylipin metabolism (Reverberi et al., 2010).

In this study, we investigated the functions of endogenous LDS1-derived oxylipins in affecting the biology of the maize pathogen *F. verticillioides* and its interaction with the host maize plant. To this purpose, we created a ΔFv*lds1* strain (*LDS1-deleted)* and we studied its phenotype, secondary metabolism and virulence. Moreover, we explored the possibility that oxylipins—acting as chromatin modifiers—may control the expression of fumonisin biosynthetic genes (FUM) and, in turn, fumonisin synthesis. Results indicate that *LDS1* deletion changes the whole oxylipin and FB-production profile of *F. verticillioides*. We here suggest that oxylipins may induce these alterations by controlling gene transcription also through a direct influence on the chromatin status.

## Materials and methods

### Fungal strains and media

The WT strain deposited as ITEM 10027 in the collection of the Institute of Sciences of Food Production (ISPA-CNR, Bari, Italy; http://server.ispa.cnr.it/ITEM/Collection) was isolated from maize in Northern Italy and maintained at −80°C. The V8 juice agar with 100 ppm of hygromycin (200 mL of V8 juice, 3 g of CaCO_3_ and 20 g agar per L) was used for the first step of mutant selection. Monoconidial cultures of all strains were obtained and cultivated in liquid media in the dark and at 25°C on a rotary shaker (150 rpm) for 7 days unless noted otherwise.

Fungal growth, conidiogenesis and spore production were determined by inoculating all the tested strains into Potato Dextrose Broth (PDB) liquid medium at 25°C. Since PDB is not conducive for FB production, we also used Czapek medium amended with cracked maize 2 g/L (abbreviated CDYM) which, as reported (Scala et al., 2013), induces FB production. CDYM (50 mL) was inoculated with 1 × 10^6^ conidia/mL. Cultures were incubated up to 15 days after inoculation (DAI) in the dark at 25°C.

### Molecular biology techniques

#### Gene deletion, fungal transformation and selection of transformants

To generate the construct for disrupting the *F. verticillioides* FVEG_09294.3 gene (putative *LDS1*) by targeted homologous recombination, specific oligonucleotides were designed to amplify the upstream (primers Lds_for_PciI and Lds_rev_StuI, presenting a *Pci*I and *Stu*I site respectively) and downstream (primers Lds_for_NaeI and Lds_rev_SbfI, presenting a *Nae*I and *Sfb*I site respectively) flanking region of *LDS1* (Supplementary Table 1). The amplicons (about 1.0 Kb each) were initially T/A cloned in the pGEM-T Easy vector and then sub-cloned, alongside with the hygromycin cassette (*Hph* box) derived from the hygromycin B resistance gene, into the vector pAN7.1 (6.7 Kb) previously digested with *Pci*I–*Stu*I at 5′ and *Nae*I–*Sbf*I at 3′. WT protoplasts were transformed with the resulting disruption cassette. Fifty hygromycin-resistant colonies were collected and transferred onto Potato Dextrose Agar (PDA) plates supplemented with 100 μg/mL of hygromycin B (Duchefa Biochemie, Haarlem, NL). Monoconidial cultures of all resistant mutants were obtained and preliminarily screened by PCR using the primer pair panlds1F-panldsR that amplified in the vector used for the deletion (Supplementary Table 1). Finally, 20 colonies were selected and further sub-cultured. The stability of these transformants was tested by two additional single-spore transfers on non-selective, and then again on selective, media. The deletion of the gene was tested by Southern blot hybridization (see dedicated paragraph).

The ΔFv*lds1* complementation strains (COM) were generated by transformation of the Fv*lds1*-deleted mutant with the WT *LDS1* allele using the geneticin resistance gene, *GenR*, as a selectable marker as described elsewhere (Gruber et al., 2012). The selection and purification of the putative LDS1-complemented strains was performed as described above concerning the generation of deleted mutants, i.e., by transferring the transformants individually onto PDA plates amended with 300 μg/mL geneticin (G418, Sigma-Aldrich, St Louis, USA). Cassette integration was verified by PCR using primers nptII_for and nptII_rev designed in the *GenR* box (*nptII*) (Supplementary Table 1).

#### Southern blot hybridization

Genomic DNA (10 μ g), extracted as previously described (Scala et al., 2013) from WT *F. verticillioides* ITEM 10027 and ΔFv*lds1* strains, was digested with *Eco*RI (10 U) at 37°C for 4 h (Fermentas). *Eco*RI-restricted DNA fragments were separated by electrophoresis and then blotted onto Hybond-N^+^ nylon membrane (Roche). Fluorescent DNA probes [Fv*LDS1* (0.8 Kb) and *Hph* (1.0 Kb)] were prepared according to the PCR digoxigenin (DIG)-labeling method (Roche) by using primers Lds_1 F and Lds_1 R (Supplementary Table 1; *Eco*RI did not restrict the 800 bp-probe fragment). The membranes were hybridized for 12–16 h in DIG-easy hybridization buffer (Roche) containing 250 ng of digoxigenin (DIG)-labeled Fv*LDS1* or *Hph* probes at 65°C.

#### Transcript quantification assay

For the *in vitro* experiments, total RNA from the mycelia of *F. verticillioides* WT, ΔFv*lds1*D, and COM strains was extracted as reported (Scala et al., 2013) at 0, 2, 5, 7, 10, and 15 DAI in CDYM cultures and used to develop reverse-transcriptase quantitative PCR (RT-qPCR) assays for Fv*LDS1* (FVEG_09294.3), Fv*LDS2* (FVEG_12540.3), Fv*LDS3* (FVEG_11670.3), and Fv*LOX* (FVEG_09897.3). For the *in vivo* experiments, total RNA from maize cobs infected and non-infected by *F. verticillioides* WT and ΔFv*lds1*D was extracted as reported (Scarpari et al., 2014) at 0, 2, 5, 7, 10, and 15 DAI. Related cDNA was used to develop RT-qPCR experiments for Fv*LDS1*, Fv*LDS2*, Fv*LDS3*, Fv*LOX*, Zm*LOX3* (AF329371), and Zm*PR4* (AJ969166). The sequence of the primers used for the RT-qPCR assays are reported in Scala et al. (2013), Brodhagen et al. (2008) and in Supplementary Table 1. Reverse transcriptase reactions were performed as previously described (Scala et al., 2013). Gene expression in the WT, ΔFv*lds1*D, and COM strains, and in maize cobs, was calculated by using the 2^−ΔΔCt^ method, i.e., by normalizing transcript levels of the gene of interest onto the transcript of a housekeeping gene [β-Tubulin for *F. verticillioides* (FVEG_05512.3) (Scala et al., 2013) and α-Actin for *Zea mays* (DQ492681.1) (Supplementary Table 1)] and onto their value at the time of inoculation (time zero). The software for relative expression quantification provided with the Line GeneK thermocycler (Bioer, PRC) was used.

#### Chromatin immunoprecipitation (ChIP)

The ChIP procedure was performed as described by the manufacturer of the EZ ChIP chromatin immunoprecipitation kit (Upstate, Charlottesville, VA). *F*. *verticillioides* WT and ΔFv*lds1*D strains were cultured in CDYM up to 15 DAI to induce FB production. Protein/DNA cross-linking was performed at harvest by incubating the cultures for 10 min in 1% formaldehyde (final concentration in the growth medium). The cross-linking reaction was stopped by addition of 0.125 M Glycine (final concentration). The mycelium was rinsed in PBS (20 mM Na_2_HPO_4_/KH_2_PO_4_ and 150 mM NaCl, pH 7.5) and then frozen in liquid nitrogen. Frozen mycelium (0.5 g) was ground in liquid nitrogen with a mortar and pestle, and the powder was resuspended in 2 mL of ice-cold DNA extraction buffer (Upstate) according to the manufacturer's instructions. Chromatin was sheared by sonication in a Hewlett-Packard V14 with 5 pulses of 60 s each at full power. Cross-linked DNA and proteins were immunoprecipitated with antibodies against hyper-acetylated histone H4 (Penta; Upstate) on the cross-linked DNA. Chromatin was then washed and eluted, and cross-links were reversed according to the manufacturer's instructions. Quantification was performed by qPCR under conditions that mirrored those for RT-qPCR but with immunoprecipitated DNA as a template (samples normalized to fresh weight). The primers used here are designed on the *FUM1* promoter region (Visentin et al., 2012) (Supplementary Table 1). We quantified the abundance of target DNA by the relative standard curve method with β-*TUB* as the endogenous reference for normalization. Two biological and three analytical repeats were performed and standard errors were calculated. Calibration curves were calculated by linear regression.

#### Sequencing analysis

Paired-end libraries with an average insert size of 500 bp were produced and sequenced with the Illumina HiSeq 1500 technology at Genomix4life (Baronissi, Italy). On average, 15 × 10^6^ reads were obtained for each *F. verticillioides* strain with a read length of 100 bp. A homemade pipeline called SUPERW (Simply Unified Pair-End Read Workflow) was developed to create a dynamic and fast tool to analyze the variation data produced from the re-sequencing experiments. The SUPERW pipeline is divided in three steps: the filtering and mapping, the variation calling and the output of the data. The first step automatically recognizes the quality of the samples redirecting them either to filtering or directly to mapping. Filtering of the reads creates a new high-quality subset of reads suitable for mapping analyses. After the filtering step, all the reads are mapped against a reference genome using the *bwa mem* (Li et al., 2009) algorithm and allowing the user to select all the possible *bwa* options. The mapped files are filtered for PCR duplicates, compressed in *bam* files, sorted and indexed (Li et al., 2009) creating as output a *bam* file from each mapping and a text file with the statistics about the trimming and the mapping steps. The mapped *bam* files and the reference genome are the input files for the variation-calling step of the pipeline.

#### Calling large structural variations

A depth of coverage approach was used to extract the large structural variations (CNVs) using the CNVnator tool (Abyzov et al., 2011), a method based on combining the established mean-shift approach to broaden the range of discovered CNVs. All CNVs were filtered for read-depth (RD) of ± 0.5 and *p*-value of 0.001. Finally the CNVs falling in the *LDS1* region were extracted and analyzed with a window approach of 100 bp to better study the “gene deletion” event.

### Phenotypic characterization of *F. verticillioides* strains

#### Fungal growth in vitro and spore germination

Fungal growth was measured as dry weight (d.w.) by weighing the mycelium after filtration (Millipore filters, 0.45 μm) and drying for 48 h at 80°C. To evaluate spore germination rates, 1 × 10^5^ spores/mL of *F. verticillioides* were inoculated in PDB and the percentage of germinated spores was estimated at 25°C 24 h after inoculation.

#### Mating-type identification

The mating types (MAT-1 and MAT-2) of *F. verticillioides* WT and mutant strains were identified by PCR using the primers fusALPHArev—fusALPHAfor for MAT-1, and fusHMGfor—fusHMGrev for MAT-2 as described (Kerenyi et al., 2004). The annealing temperature for hybridization of both primers was adjusted to 56°C. The amplicons of the *MAT-1* and *MAT-2* idiomorphs are approximately 200 and 260 bp, respectively. For the definition of inter-strain fertility and ability to produce perithecia, the general crossing protocol was applied as described by Leslie and Summerell (2006). Reference strains obtained from the official collection of ISPA-CNR, Bari, Italy, were used: ITEM 15575 (KSU A-149) for MAT-1 and ITEM 15574 (KSU A-999) for MAT-2 respectively. Strains of the female parent were inoculated onto Petri dishes (Ø 9 cm) containing carrot agar, and the strain serving as the male was inoculated on Petri dishes (Ø 5 cm) containing water agar on the same day. The strains were incubated at 25°C in the dark. After 7 days, conidia from the male parent were suspended in 5 mL of 0.25% Tween®60 (Sigma-Aldrich, St. Louis, MO, USA) solution and 1 mL of the conidial suspension was spread on the surface of the female culture and worked into the mycelia with a L-shape rod. Fertilized plates were then incubated at 25°C in darkness and weekly checked for perithecia formation up to 8 weeks after the crossing. Crossings were repeated 5 times for each fungal strain and considered positive when perithecia were found in at least 2 out of 5 crossing trials and as negative when perithecia were absent on at least 3 out of 5 crossing trials (Leslie and Summerell, 2006).

#### Virulence assays on artificially inoculated maize cobs

WT and mutant *F. verticillioides* strains were inoculated on Potato Dextrose Agar (PDA) plates and incubated at 25°C in darkness for 1 week; then, conidial suspensions were prepared by adding 5 mL of sterile water on each colony. Suspensions were adjusted to a concentration of 10^5^ conidia/mL and used to infect cobs of a commercial maize hybrid. These were collected at dough stage and infected with the pin bar technique. Wounds were made all around the middle area of the cob, without leaf removal, obtaining three portions with visible holes. After inoculation with 10^4^ conidia per wound, cobs were placed in plastic bottles containing 50 mL of Hoagland's solution at the bottom (1 mL KH_2_PO_4_ 1 M, 5 mL KNO_3_ 1 M, 5 mL Ca(NO_3_)_2_ × 4H_2_O 1 M, 2 mL MgSO_4_ × 7H_2_O 1 M and sterile water to 1 L) as nutritional source (Hoagland and Arnon, 1950) and incubated at RT. Non-inoculated cobs were included. We performed our experiments under environmental conditions conducive for *F. verticillioides* infection. In our lab, room temperature during summer is stable around 23–25°C and there is natural light with natural day/night duration. Even if these may not be the most conducive conditions possible, they are largely permissive, as demonstrated in previous works (Marin et al., 2010). Different incubation times were considered (0, 2, 4, 7, and 15 DAI) and each thesis was managed in triplicate. After incubation, cobs were stored at −18°C until analysis. After hand de-husking, the severity of the ear attack by WT and ΔFv*lds1*D strains of *F. verticillioides* was evaluated using a visual rating scale including seven classes based on the percentage of visibly infected kernels (Disease Severity Rating-DSR: 1 = 0%-no infection; 2 = 1–3%; 3 = 4–10%; 4 = 11–25%; 5 = 26–50%, 6 = 51–75%; 7 = 76–100%) as reported (Reid et al., 2002). Visual rating is a common practice for in-field evaluation of infection severity. The use of this kind of scales has been reported on detached maize cobs in several papers (Reid et al., 2002 and reference therein) as they are quite effective in representing maize infection severity. In our case, also because infection was the outcome of artificial inoculation under controlled conditions, cob symptoms were only due to WT or ΔFv*lds1*D-mutant *F. verticillioides* strains, given that control cobs were clear of infection symptoms.

In order to monitor fungal growth into the maize cobs, a specific SYBR green qPCR method was set by using *FUM1* primers (Supplementary Table 1). Real-time PCR was prepared in a 20 μ L reaction mixture containing SYBR green JumpStart Taq Ready as described in Reverberi et al. (2013). Standard calibration was performed plotting the Real-time PCR signals obtained for *F. verticillioides* genomic DNA in the concentration range 1 pg–100 ng. The equation describing the increase of DNA concentration was calculated (*y* = −0,9754x + 28,591, *R*^2^ = 0.991) and used afterwards as a reference standard for the extrapolation of quantitative information of DNA targets of unknown concentrations. The final amount of fungal DNA (ng) was referred to 1 μ g of total DNA. The efficiency of the PCR reaction (101%) was obtained from the calibration curve slope (*E* = 10−1 / slope − 1).

### Metabolite analyses

#### Oxylipins

Oxylipins were extracted as described by Scala et al. (2013) with slight modifications. Samples were analyzed by liquid chromatography (HPLC 1200 series rapid resolution, Agilent Technologies, Santa Clara, CA, USA) coupled to triple quadrupole (G6410A series triple quadrupole, QqQ, Agilent Technologies, CA, USA) equipped with an electrospray ionization (ESI). Experiments in MRM in negative ion mode [M-H]^−^ were performed and data processed as reported by Ludovici et al. (2014).

#### Free and total fumonisins

Free and total fumonisins were determined according to our previous work (Dall'Asta et al., 2010). Fumonisins obtained after sample hydrolysis were measured as the sum of hydrolyzed FB_1_, FB_2_, and FB_3_. All of the results are expressed as the sum of FB_1_, FB_2_, and FB_3_ equivalents, considering a correction factor due to the different molecular weight of parent and hydrolyzed compounds, and referred to as “total fumonisins” (FB_tot_).

#### Statistical analysis

Data are presented as the mean value (±SE) of three independent measurements from two separate experiments. In each experiment, data sets were pooled and compared using Mann-Whitney's test and the differences were considered significant when the *p*-value was <0.05.

## Results

### Molecular profile of Fv*lds*1-deleted *F. verticillioides*

Our previous study (Scala et al., 2013) identified the *bona fide LDS1* gene homolog as one of the putative oxylipin-forming genes most highly expressed during the saprophytic growth of WT *F. verticillioides* (ITEM 10027) on a FB-inducing medium containing cracked maize. To study its function in *F. verticillioides*, the Fv*LDS1* gene (acc. N. FVEG_09294.3 as annotated on the reference, sequenced strain 7600, see below) was targeted for deletion to create ΔFv*lds1* mutants. Putative deleted strains were PCR-screened and those, which demonstrated clear molecular differences in comparison with the WT were selected (data not shown). One out of 20 PCR-selected mutants was further characterized and the genomic organization of Fv*LDS1* was studied by Southern blot hybridization using fragments of *LDS1* and *Hph* as molecular probes. The results indicated the presence of two hybridization signals with the *LDS1* probe whilst at least one putative mutant (thereafter named ΔFv*lds1*D) presented a recognizable deletion pattern (Supplementary Image 1). The same mutant strain carried the *Hph* box in its genome (Supplementary Image 1).

At least three LDS-encoding genes—namely, *LDS1, LDS2*, and *LDS3*—are present in the genome of *F. verticillioides* strain 7600 released by the Broad institute (http://www.broadinstitute.org/annotation/genome/fusarium_group/MultiHome.html). *LDS2* and *LDS3* are quite dissimilar in their nucleotide sequence from the *LDS1* hybridization probe (32 and 29% identity, respectively). Thus, it may be assumed that the hybridization pattern in Supplementary Image 1 is indicative of the presence of two *LDS1* paralogs, *LDS1a*, and *LDS1b* in our WT strain. Since only one Fv*LDS1* gene is reported in the genome of *F. verticillioides* reference strain 7600, we sequenced our WT strain (http://www.ncbi.nlm.nih.gov/bioproject/263314) as well as its mutant ΔFv*lds1*D for pinpointing any difference with the reference strain. A depth-of-coverage approach was used to search for copy number variation (CNV) of genes. Mapped files were used to call CNV regions. Two thousand and twenty-six CNV regions were identified; among them, only those with a minimum read depth (RD) of 1.5 and a *p*-value of 0.05 were considered. In this way, 1407 total CNVs (695 duplications and 713 deletions) were identified. By comparing the ΔFv*lds1*D and WT strains against the genome of the reference strain, we established a significant CNV in the *LDS1* locus (genomic region Supercontig_3.12 coord. 1,190,484 to 1,187,862 reverse strand). In particular, the comparison between our WT and the FV7600 reference strain showed the presence of a massive duplication (~24 Kb; RD 1.8) in the *LDS1* locus of our WT genotype (Supplementary Image 2A). The comparison of ΔFv*lds1*D genome against the genome of the reference strain (FV7600) did not show differences in the depth of coverage (Supplementary Image 2B). These results can be explained with the presence of two *LDS1* genes in the WT and a consequent deletion of one copy (thereafter named *LSD1b*) in the mutant strain ΔFv*lds1*D, confirming the Southern blot results.

*LDS1* function was recovered in the deleted genetic background of ΔFv*lds1*D by ectopic expression of the WT *LDS1b* genomic sequence under the control of the endogenous promoter. Insertion of the complementation cassette was verified by PCR of the *GenR* box (*nptII*). The expected 447-bp fragment was amplified in 2 out of 5 putative complemented strains (COM) (Supplementary Image 3).

### *In vitro* phenotypic analysis of F*vlds1*-deleted *F. verticillioides*

**Figure 1:**
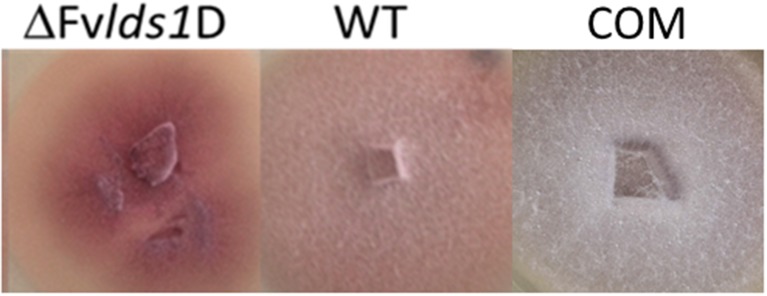
**Phenotype characterization**. Phenotype of *F. verticillioides* WT, ΔFv*lds1*D, and COM strains grown in PDA medium 7 DAI.

The morphological features and the spore differentiation ability of the ΔFv*lds1*D strain and of WT strain were further characterized (Figure 1). The most obvious differences with the WT were related to the mycelium network consistency (fluffy in the WT, thin and leathery in the ΔFv*lds1*D strain), in the color (pink in the WT and deep pink in the ΔFv*lds1*D strain) and in the growth mode on solid medium (aerial in the WT vs. submerged in the ΔFv*lds1*D strain). The COM strain recovered the morphological features of the WT strain (Figure 1).

In liquid media, the deleted strain grew faster than the WT and COM at 15 DAI (Table 1) whereas in solid media its growth rate was similar (data not shown). ΔFv*lds1*D produced more conidia—which germinated significantly faster—than the WT and COM, starting 24 h after inoculation at 25°C (Table 1). These results demonstrate that the deletion of *LDS1b* significantly affected colony morphology, growth rate on liquid medium (*p* < 0.001), conidia production (*p* < 0.01) and their germination speed (*p* < 0.05) in *F. verticillioides*.

**Table 1:** **Phenotypic characterization of oxylipin-defective ΔFv*lds1*D *in vitro***.

**Fungal strain**	**Growth mg d.w./mL**	**Conidia/mL**	**Spore germination (%)**
ΔFv*lds1*D	740 ± 82	8.4 × 10^7^ ± 1.1 × 10^6^	100.0 ± 5.2
WT	400 ± 45	6.4 × 10^7^ ± 5.6 × 10^6^	81.4 ± 3.1
COM	425 ± 74	6.8 × 10^7^ ± 1.9 × 10^6^	79.5 ± 6.4

Growth (mg d.w./mL), conidiogenesis (conidia/mL), and conidia germination (%) of F. verticillioides WT, COM, and ΔFvlds1D strains in PDB medium. Results represent the mean of n = 6 analyses deriving from two biological replicates each repeated three times ± SE.

Preliminary but suggestive results were obtained in sexual crossing experiments. We firstly identified the mating type of our strains (MAT-1; Supplementary Image 4) and then we crossed them with the reference strain for MAT-2, as suggested by Leslie and Summerell (2006). Both the WT and ΔFv*lds1*D were able to produce perithecia in at least two independent experiments, even if with different efficiencies (number of fertile crosses/number of total crosses: WT-100%; ΔFv*lds1*D-83%, Supplementary Image 4).

### Oxylipin biosynthesis *in vitro*: gene expression and mass spectrometry analysis

**Figure 2:**
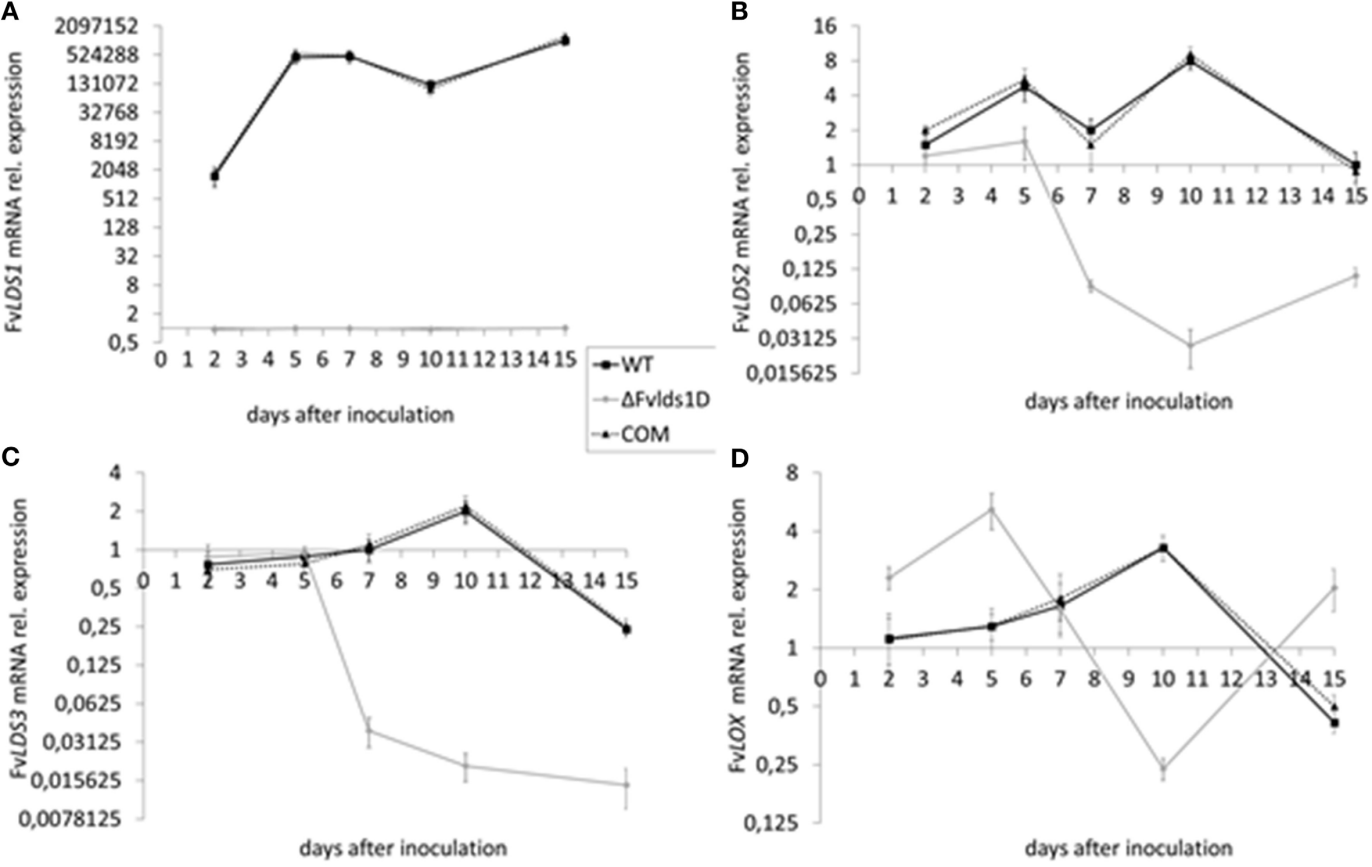
**mRNA levels of *LDS1-3* (A–C) and *LOX* (D) at 2-15 DAI**. WT, mutant (ΔFv*lds1*D) and COM strains of *F. verticillioides* were grown in FB-inducing CDYM. Relative mRNA expression is calculated by using the 2^−ΔΔCt^ method, i.e., by normalizing gene of interest expression onto housekeeping gene expression and to their value at the time of inoculation (time zero). *LDS1a* and *b* were not discriminated in this analysis. Results are the mean (± SE) of six replicates from two independent experiments.

As stated previously, at least four *LDS* genes (*LDS1a* and *b*, *LDS2*, and *LDS3*) and one gene encoding a putative 13*S*-LOX (based on its high similarity with *LOX2* in *F. oxysporum*, Brodhun et al., 2013) are present in our *F. verticillioides* WT strain. We tested their transcript level in the WT, ΔFv*lds1*D and COM strains grown on CDYM by RT-qPCR. All *LDS* genes were strongly down-regulated in ΔFv*lds1*D (Figures 2A–C; *LDS1a* and *b* were not discriminated in this analysis). It is also worth mentioning that the expression of the putative 13*S*-*LOX* gene, instead, was significantly up-regulated early after inoculation (2 and 5 DAI) when compared to the WT (Figure 2D). Transcript levels of oxylipin genes in the COM strain were similar to the WT (Figures 2A–D).

In order to quantify the effect of this general re-modulation of the expression of oxylipin-related genes in the ΔFv*lds1*D strain, a set of 17 oxylipins derived from both linoleic and linolenic acid was analyzed (Table 2; Supplementary Data Sheet 1). The oxylipin set was divided accordingly to its putative enzymatic origin (specifically, if LDS- or LOX-derived) following a published protocol (Strassburg et al., 2012). The oxylipin profile originated by the comparison between ΔFv*lds1*D and the WT strains partly confirmed RT-qPCR results reported in Figures 2A–D. The COM strain recovered the oxylipin profile of the WT strain (Table 2).

**Table 2:** **MRM quantification of oxylipins under *in vitro* experiment**.

	**10-HODE**	**11-HPODE**	**12,13-diHOME**	**12-epoOME**	**9,10-diHOME**	**9-epoOME**	**8,13-diHODE**	**8-HPODE**	**8-HODE**
**LDS-DERIVED OXYLIPINS (μM)**									
WT	25,3 ± 2,5	2,5 ± 0,5	1,2 ± 0,3	10,8 ± 0,5	2,3 ± 0,4	22,8 ± 2,5	175,3 ± 12,4	1,6 ± 0,1	33,1 ± 3,5
ΔFv*lds1*D	30,6 ± 3,5	0,3 ± 0,1	0,7 ± 0,1	4,5 ± 0,5	1,3 ± 0,3	11,7 ± 1,5	0,7 ± 0,2	<LOQ	9,3 ± 1,5
COM	23,5 ± 2,4	2,1 ± 0,4	1,3 ± 0,1	11,5 ± 2,1	2,0 ± 0,5	23,5 ± 3,5	178,2 ± 18,5	1,8 ± 0,4	32,2 ± 4,5
	**13-HODE**	**13-HPODE**	**13-oxoODE**	**13-HOTrE**	**9-HODE**	**9-HPODE**	**9-oxoODE**	**9-HOTrE**	
**LOX-DERIVED OXYLIPINS (μM)**									
WT	8,5 ± 0,5	2,2 ± 0,5	4,6 ± 0,7	0,6 ± 0,2	119,8 ± 22,1	2,4 ± 0,5	4,9 ± 0,6	0,5 ± 0,2	
ΔFv*lds1*D	3,4 ± 0,5	0,2 ± 0,1	<LOQ	0,6 ± 0,1	52,8 ± 2,5	0,3 ± 0,1	0,1 ± 0,1	0,5 ± 0,1	
COM	9,2 ± 0,6	2,5 ± 0,2	5,2 ± 0,8	0,8 ± 0,4	120,4 ± 11,5	3,0 ± 0,4	5,2 ± 0,7	0,6 ± 0,2	

Quantification of 17 oxylipins (μM) in mycelia of F. verticillioides WT, ΔFvlds1D and its complementation mutant (COM), 7 DAI under in vitro conditions (see Materials and Methods Section).

The accumulation trend for the whole set of oxylipins was followed from 1 up to 15 DAI (Supplementary Data Sheet 1). Table 2 summarizes the results of 7 DAI, i.e., when the difference between the strains is more marked. Putative LDS1 products, specifically 8-HPODE, 8,13-diHODE and 8-HODE, were severely down-represented in the ΔFv*lds1*D mutant as compared to the WT, while 10-HODE was up-regulated in the mutant strain. Oxylipins in the 9- and 13-LOX pathways (i.e., HPODE, HODE, and oxoODE) were also affected in ΔFv*lds1*D (Table 2).

### Fumonisin B production and gene expression

**Figure 3:**
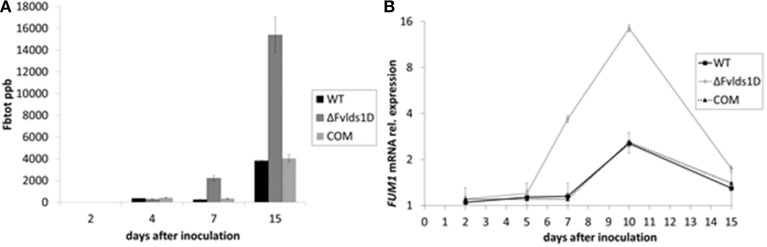
**Fumonisin production and transcript of the key biosynthetic gene *FUM1 in vitro***. **(A)** Quantification of the main B-series fumonisins (FB_tot_ = FB_1_ + FB_2_ + FB_3_, expressed as ppb) and **(B)** *FUM1* mRNA quantification in the WT, mutant (ΔFv*lds1*D) and COM strains of *F. verticillioides* relative to inoculation time 0. Fungi were grown for 2–15 DAI in FB-inducing CDYM; results are the mean (± SE) of six replications deriving from two independent experiments.

The main B-series fumonisins (FB_tot_ = FB_1_, FB_2_, and FB_3_) were quantified in WT, COM and ΔFv*lds1*D strains at 2–15 DAI, i.e., at the peak of toxin production under our experimental conditions. ΔFv*lds1*D produced significantly (*p* < 0.001) more FB than the WT and COM strains starting from 7 DAI onwards (Figure 3A), with a comparable effect on FB_1_, FB_2_, and FB_3_ (data not shown). The fumonisin B increase was apparently modulated at transcriptional level. In fact, *FUM1* was more expressed at 7 and 10 DAI (*p* < 0.001) in the ΔFv*lds1*D strain compared to the WT as well as to the COM strain (Figure 3B).

### Effects of *LDS1b* deletion on growth and virulence of *F. verticillioides* on maize cobs

**Figure 4:**
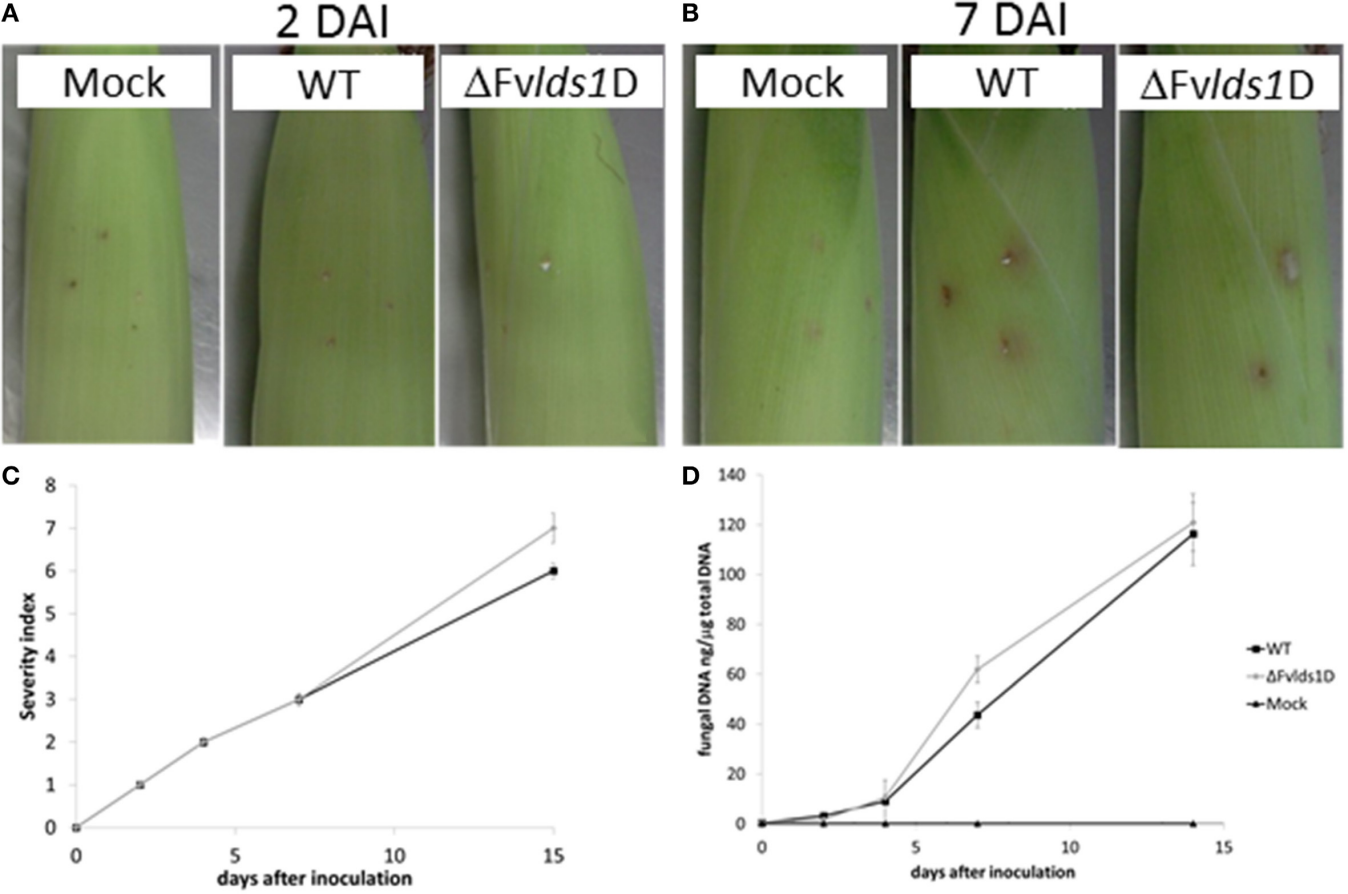
**Virulence analysis of the two strains of *F. verticillioides* (WT and ΔFv*lds1*D mutant)**. Visual appearance of cobs artificially wounded, non-inoculated (mock) and inoculated with the two strains of *F. verticillioides* (WT and ΔFv*lds1*D) **(A)** 2 and **(B)** 7 DAI at 25°C in bottles containing Hoagland's solution. **(C)** Severity of *F. verticillioides* ear attack evaluated by a visual rating scale based on the SCIA (wounding and non-wounding) method (Marin et al., 2010). **(D)** Abundance of fungal DNA in maize ears at different DAI, as a measure of tissue colonization. This method adopts 7 classes based on the percentage of visibly infected kernels (Disease Severity Rating: 1 = 0%-no infection; 2 = 1–3%; 3 = 4–10%; 4 = 11–25%; 5 = 26–50%, 6 = 51–75%; 7 = 76–100%).

Oxylipins play a significant role in fungal virulence and in the cross talk with the host (Tsitsigiannis and Keller, 2007; Brodhun et al., 2009; Christensen and Kolomiets, 2011; Scarpari et al., 2014). To assess the virulence phenotype of the ΔFv*lds1*D strain, we inoculated maize cobs with the mutant or the WT strain. Rapid mold development was noticeable for the ΔFv*lds1*D mutant, slightly slower for the WT. Cobs wounded but not inoculated did not show any sign of fungal growth (Figures 4A,B). The severity of ear rot disease symptoms was rated by a published index (Reid et al., 2002). As shown in Figure 4C, the mutant strain caused more damage than the WT at 15 DAI. qPCR aimed at quantifying fungal growth into plant tissues confirmed this result. The mutant indeed colonized the host tissue significantly faster (*p* < 0.01) than the WT strain at 7 DAI, whereas this difference was lost at 15 DAI (Figure 4D).

### Oxylipin formation *in vivo*: gene expression and mass spectrometry analysis

**Figure 5:**
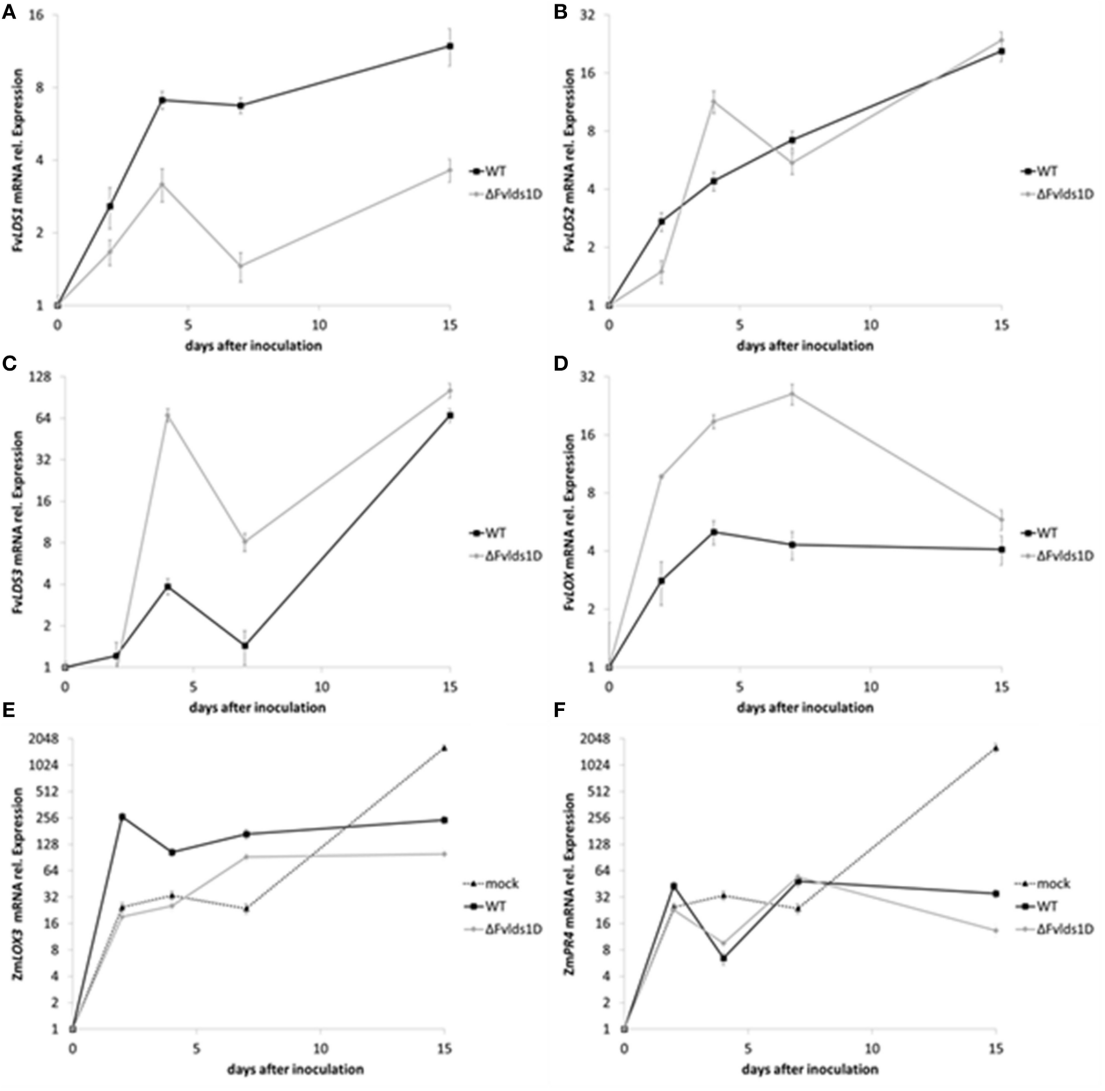
**(A–F)** mRNA levels of fungal and plant genes in infected cobs. *F. verticillioides LDS1* **(A)**, *LDS2* **(B)**, *LDS3* **(C)**, and *LOX* **(D)**, *Zea mays LOX3* **(E)**, and *PR4* **(F)** were quantified at 2–15 DAI in maize cobs non-inoculated (mock), or inoculated with the WT or mutant strain (ΔFv*lds1*D). Relative mRNA expression is calculated by using the 2^−ΔΔCt^ method, i.e., by normalizing gene of interest expression onto housekeeping gene expression and to their value at the time of inoculation (time zero). Results are the mean (± SE) of six replications deriving from two independent experiments.

Fungal pathogens as well as host plants produce oxylipins during the interaction. The shared chemical structure is at the basis of their mutual ability to affect defense onset (in the host) and virulence (in the fungus) (Christensen and Kolomiets, 2011). We analyzed the expression of some fungal oxylipin-related genes and one of the most studied maize genes (Zm*LOX3*) coding for LOX enzymes and known to be important in the interaction of maize with other mycotoxin-producing fungi (Gao et al., 2009). In particular, we monitored the expression of fungal *LDS1*-*3* and *LOX* as well as of maize Zm*LOX3* (Figures 5A–E) in maize cobs artificially inoculated. As reported in Figure 5A, total Fv*LDS1* transcript (*LDS1a* plus *b*) is conspicuously less abundant, but not completely absent, in the ΔFv*lds1*D strain as compared to the WT. Since we deleted only one out of the two *LDS1* copies present in the genome of our parental strain, the results suggest that the non-deleted copy *LDS1a* may be expressed *in vivo* (Figure 5A) while it is not *in vitro* (see Figure 2A for comparison). The transcript abundance of other oxylipin-related genes was similar (*LDS2*, Figure 5B) or higher (*LDS3, LOX*, Figures 5C,D) in the ΔFv*lds1*D strain when compared to the WT. It is noteworthy that while *LDS2* and *LDS3* were down-regulated under *in vitro* conditions at all time-points (*p* < 0.01), both are up-regulated *in vivo*, likely upon the influence of the host tissues. Conversely, Fv*LOX* expression was consistently up-regulated in the ΔFv*lds1*D strain as compared to the WT (*p* < 0.001), even more conspicuously than under *in vitro* conditions (see Figure 2D for comparison).

Maize up-regulated Zm*LOX3* in the early stage of fungal infection (up to 7 DAI; *p* < 0.01). Then, at 15 DAI, i.e. when the pathogen achieved its maximum development in the host tissues, Zm*LOX3* was significantly (*p* < 0.001) repressed both upon WT as well as ΔFv*lds1*D infection (Figure 5E) as compared to uninfected maize. Despite this shared trend, significant differences emerged between the two *F. verticillioides* strains. Namely, the Zm*LOX3* transcript was more abundant in the WT- than the ΔFv*lds1*D-infected maize tissues (*p* < 0.001).

Maize defense responses include activation of class 4 pathogenesis-related (PR) proteins. PR4 proteins are chitinases contributing to the degradation of the fungal cell wall (Wang et al., 2011); ethylene- and jasmonic acid-related pathways (Van Loon et al., 2006) control PR4 expression in *Arabidopsis*. As jasmonic acid is an oxylipin, and given the broad-spectrum alteration of the oxylipin profile in pathogen-challenged maize cobs observed in this and in recent works (Christensen et al., 2014; Scarpari et al., 2014), we monitored Zm*PR4* expression as a marker of plant defense responses triggered by oxylipins. The infection by both *F. verticillioides* strains (WT and ΔFv*lds1*D) modulated Zm*PR4* expression in a trend intriguingly similar to that of Zm*LOX3* (Figures 5E,F in comparison). Both Zm*PR4* and Zm*LOX3* expression were sharply up-regulated (to a level comparable to the infected cobs) in the mock-inoculated cobs at early time-points, indicating that wounding itself induces their expression as reported in other studies (Wang et al., 2011). However, the transcript levels of both genes were seemingly kept at bay by the invading fungus at later time-points, and more efficiently by the ΔFv*lds1*D mutant than by the WT. Specifically, Zm*PR4* expression was up-regulated at 15 DAI even if significantly (*p* < 0.01) less in the mutant- than in the WT-infected tissues (Figure 5F). Therefore, as whole, *in vivo* experiments suggest that Zm*LOX3* as well as Zm*PR4* were affected consistently and similarly by pathogen invasion, and that the ΔFv*lds1*D strain triggers plant defense responses less vigorously than the WT.

To validate the RT-qPCR results on the expression of the oxylipin-related genes at the metabolic level, we quantified 15 different oxylipins in the WT- and ΔFv*lds1*D-infected and non-infected cobs at 2–15 DAI (Table 3; Supplementary Data Sheet 2). Almost every oxylipin monitored achieved its maximum level at 15 DAI (Supplementary Data Sheet 2). In Table 3, their amount is reported at 15 DAI in maize cobs non-infected (mock), infected with the WT or ΔFv*lds1*D strain. Firstly, it can be pinpointed that the only oxylipins consistently down-modulated in the ΔFv*lds1*D compared to the WT-infected samples (both *in vitro*—see Table 2—and *in vivo*, Table 3) were 8-HPODE, 8,13-diHODE and 8-HODE. This may be because they are the main putative products of fungal LDS, so their synthesis would be directly affected by *LDS1*b deletion. Intriguingly, the other oxylipins (LDS- as well as LOX-derived ones) are up regulated in the maize cobs infected with ΔFv*lds1*D strain compared to those infected with the WT strain (Table 3). This result confirmed the hypothesis that oxylipin biosynthesis is tightly coordinated in *F. verticillioides* even during maize infection, although with different trends *in vitro* and *in vivo*.

**Table 3:** **MRM quantification of oxylipins under *in vivo* experiment**.

	**11-HPODE**	**12,13-diHOME**	**12-epoOME**	**9-epoOME**	**8,13-diHODE**	**8-HPODE**	**8-HODE**	
**LDS-DERIVED OXYLIPINS (μM)**								
WT	1,1 ± 0,2	0,6 ± 0,1	15,5 ± 1,2	20,6 ± 3,5	15,2 ± 2,3	0,9 ± 0,2	0,6 ± 0,1	
ΔFv*lds1*D	2,3 ± 0,2	0,7 ± 0,2	26,4 ± 3,2	27,8 ± 3,2	2,5 ± 0,6	0,2 ± 0,1	0,3 ± 0,1	
Mock	1,1 ± 0,3	0,5 ± 0,1	11,2 ± 2,2	14,7 ± 2,2	<LOQ	0,1 ± 0,05	0,1 ± 0,05	
	**13-HODE**	**13-HPODE**	**9-HODE**	**9-HPODE**	**13-oxoODE**	**9-oxoODE**	**13-HOTrE**	**9-HOTrE**
**LOX-DERIVED OXYLIPINS (μM)**								
WT	32,6 ± 4,1	0,7 ± 0,1	39,1 ± 5,2	1,2 ± 0,2	3,2 ± 0,5	1,1 ± 0,1	1,7 ± 0,2	1,9 ± 0,4
ΔFv*lds1*D	41,7 ± 3,3	1,6 ± 0,2	66,4 ± 5,5	2,6 ± 0,4	4,7 ± 0,4	2,4 ± 0,2	2,3 ± 0,1	3,3 ± 0,1
Mock	23,1 ± 4,2	1,3 ± 0,2	27,1 ± 4,3	1,4 ± 0,3	3,1 ± 0,5	1,5 ± 0,3	1,2 ± 0,4	1,1 ± 0,2

Quantification of 15 oxylipins (μM) in maize cobs infected with F. verticillioides WT, ΔFvlds1D, or non-infected (mock) at 15 DAI.

The ΔFv*lds1*D mutant strain produced significantly more FB_tot_ (*p* < 0.001) than the WT at 15 DAI in maize ears (5828 ± 458 vs. 867 ± 96 ppb). As shown in Table 3, 9-H(P)ODE is produced at higher levels in ΔFv*lds1*D-challenged maize ears than in WT-infected cobs (e.g., 66.42 vs. 39.11 μM for 9-HODE). 9-H(P)ODE is known to promote mycotoxin synthesis, specifically during the interaction between *F. verticillioides* and maize (Christensen et al., 2014). It can thus be suggested that two factors may contribute to the enhanced FB production in the mutant strain: the down-modulation of LDS products such as 8-H(P)ODE, and the enhancement of 9-H(P)ODE synthesis, possibly by ZmLOX3.

### How do oxylipins affect fumonisin synthesis?

**Figure 6:**
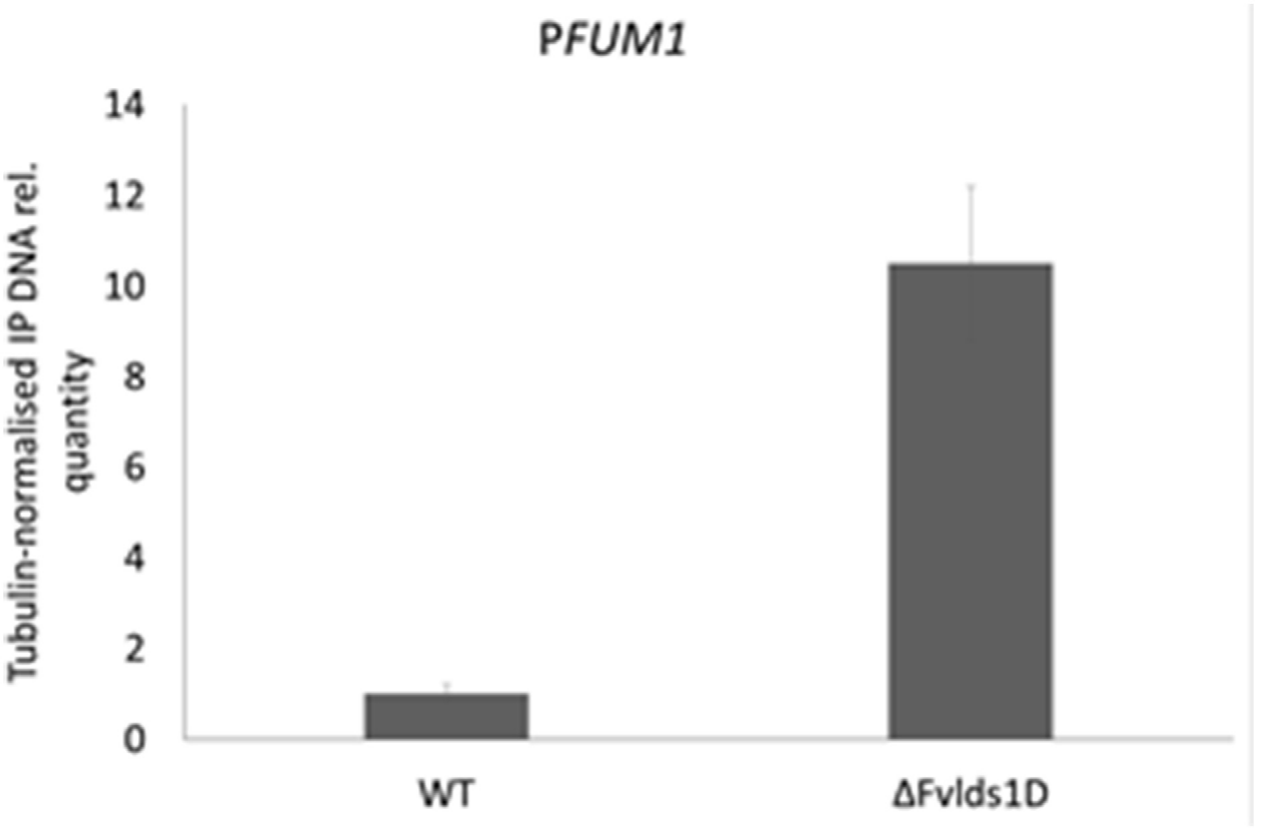
**Relative acetylation levels of the *FUM1* gene promoter (P*FUM1*) in *F. verticillioides* WT and ΔFv*lds1*D strains**. Acetylation levels were analyzed by ChIP with antibodies specific to hyper-acetylated histone H4 on DNA from fungi cultured in FB-inducing CDYM. The abundance of target DNA was quantified by the comparative CT method with β-*TUB* as the endogenous reference for normalization. Error bars indicate the standard errors of two biological and three technical repetitions.

We previously showed that FB levels are enhanced in oxylipin-defective mutants of *F. verticillioides* because of transcription activation (see FB_tot_ and *FUM1* transcript quantification in Figures 3A,B, for comparison). We speculated that this may be ascribed to altered acetylation levels of histone proteins at P*FUM1*, as reported under FB-inducing conditions for another WT strain of *F. verticillioides* (Visentin et al., 2012). To test this hypothesis, we performed a chromatin immunoprecipitation (ChIP) assay with a commercial antibody targeted to the hyper-acetylated form of histone H4 and focused our analysis on P*FUM1* of the WT and ΔFv*lds1*D strains grown in FB-inducing medium (CDYM). The quantities of immunoprecipitated DNA fragments containing P*FUM1* were significantly (*p* < 0.001) greater in the ΔFv*lds1*D than in the WT strain (Figure 6). This result suggests a role for oxylipins in modulating the expression of gene(s) in the FUM cluster at the chromatin level.

## Discussion

*F. verticillioides* is a ubiquitous colonizer of maize kernels even if its presence is often asymptomatic. The major concern related to maize ear rot is not the disease *per se* but the production of the neurotoxic and carcinogenic fumonisins (Brown et al., 2006). Moreover, the presence of conjugated fumonisins, which alone may equalize the content of free fumonisins, worsens the safety issues, being the plant matrix-complexes released in free form upon animal digestion, virtually doubling the content of mycotoxins in feed and food (Dall'Asta et al., 2010). The description of the biology of the fungus, its invasion procedures and regulation of mycotoxin biosynthesis is still under way and yet absolutely needed for an efficient control of this highly invasive and often “silent” pathogen. This study explores some molecular mechanisms linking several features of its biology. Specifically, we describe how one of the most—inter-kingdom—conserved classes of signaling molecules, the oxylipins, may control *F. verticillioides* biology. Over recent years, several studies illustrated the role of oxylipins in fungal pathogens and in plant-pathogen interactions (Tsitsigiannis and Keller, 2007; Gao et al., 2009; Reverberi et al., 2010; Christensen et al., 2014; Scarpari et al., 2014). Oxylipins may represent almost stable and yet reactive molecular species able to transduce environmental *stimuli* efficiently into transcriptional reprogramming. They control multiple aspects of fungal as well as of plant biology (Tsitsigiannis and Keller, 2007; De Geyter et al., 2012). Most recently, Kolomiets group showed that a unique monocot-specific 9-LOX of maize (Zm*LOX12*) is crucial in driving defense responses against *F. verticillioides*, possibly by indirectly affecting the synthesis of jasmonic acid (Christensen et al., 2014). Moreover, Dall'Asta et al. (2014) demonstrated that in natural field conditions, the LOX-derived 9-HODE marks the maize hybrids containing the highest amount of fumonisins. In particular, the authors demonstrated that along the different growing stages, 9-HODE became a discriminant entity for maize hybrids only at the harvest stage.

The structure of oxylipins, conserved in both the pathogen and its host, allows inter-kingdom communication and makes them play a demonstrated role in plant-pathogen interactions (Christensen and Kolomiets, 2011). In some instances, oxylipins produced by the host as part of its defensive molecular apparatus (such as 9-HPODE) may end up inducing mycotoxin synthesis rather than controlling cell death processes in the plant and pathogen (Rusterucci et al., 1999; Shlezinger et al., 2011).

Very little information is available on the role of fungal oxylipins in the plant cell. A previous study on the *A. ochraceus/Triticum durum* pathosystem indicated that the deletion of a fungal *LOX* gene (Ao*LOX1*) hides the fungus to the host. In fact, the wheat defense system does not react upon mutant strain infection (Reverberi et al., 2010). In the *A. flavus/Z. mays* interaction, the scarce production of HPODEs by the fungus enhances the capacity of the host to produce them (Scarpari et al., 2014). This means that other fungal oxylipins, such as LDS-derived products, may act as DAMPs or MAMPs and be perceived by the host surveillance system to activate defense responses (Savchenko et al., 2010). The leading hypothesis therefore is that the fungus produces its own oxylipins to drive its attack/reproductive machinery, but these compounds may end up alerting the plant defense system.

In this study, we demonstrated that LDS-derived oxylipins are involved in controlling growth, sporulation and mycotoxin production by the fungus, as well as in shaping the oxylipin profile in the producing microbe, also during its interaction with the host. In fact, even if it is almost impossible to discriminate between oxylipins produced by the host and by the pathogen, a change in the oxylipin profile can be described during the interaction—the most relevant trend being an up-regulation of the oxylipin machinery, likely in both organisms. Analogously to what recently described for the interaction between *A. flavus* and the kernels of *Z. mays* (Scarpari et al., 2014), also in our pathosystem the over-production of 9-H(P)ODE and the decrease in LDS products [such as 8H(P)ODE] may enhance fumonisin synthesis compared to kernels infected by the WT strain. The *in vivo* results confirmed the role of LDS-derived oxylipins in the pathogenic behavior of *F. verticillioides* on *Z. mays*. In spite of the lower tissue colonization rate by the WT fungus as established by visual analysis of infection and qPCR results, maize reacted more promptly to WT than to ΔFv*lds1*D invasion. The absence of *LDS1b* influenced the two-way interaction and made the pathogen trigger less the host-defense responses, confirming a role for fungal oxylipins as elicitors of plant defenses. Schenk and colleagues demonstrated indeed that, in *Arabidopsis thaliana* as well as in our pathosystem, oxylipins influence host defense and fortify resistance against pathogens (Schenk et al., 2014).

The pleiotropic effect, whereby a perturbation of oxylipin metabolism that leads to a deregulation of mycotoxin production, is similar to what reported previously in the IRT4 strain of *A. flavus* (Brown et al., 2009). This strain is silenced in all four *Ppo* genes and has dramatically increased aflatoxin production when compared to the WT. Thus, we suggest that also in *F. verticillioides* some products of *Ppo* (*LDS*) genes repress or at least down-modulate mycotoxin production. This hypothesis is supported by the significant up-regulation of the key biosynthetic gene *FUM1* in the ΔFv*lds1*D strain compared to the WT (Figure 3B). Our results would suggest that LDS1-related products might coordinate the synthesis of the other oxylipins and, in turn, re-shape *F. verticillioides* lifestyle, namely growth, aggressiveness and toxin biosynthesis.

How may oxylipins affect gene expression and cell metabolism in fungi? Recently, different hypotheses on how the cell perceive and transduce oxylipins to reprogram the cell transcriptome have been proposed. Keller's group identified G protein-coupled receptors (GPCRs) as main components in exogenous oxylipin perceiving and signaling (Affeldt et al., 2012). However, as for endogenous oxylipin perception, a nuclear receptor such as the mammalian PPARγ through which oxylipins modulate the transcription in a hormone-like fashion (Itoh et al., 2008) does not seem to exist in fungi. A recent paper proposed peroxisomal proteins such as PEX11 as potential targets for oxylipins, even if the relation with the transcriptional reprogramming of secondary metabolism has still to be demonstrated (Reverberi et al., 2012). In the present study, we explored the possibility that oxylipins may indirectly modify chromatin in order to contribute to transcriptional re-programming. Animal histone deacetylases (HDACs) and histone acetyl transferases (HATs) can be post-translationally modified by carbonylation through direct reaction with phlogogenous oxylipins such as prostaglandins (Ravindra et al., 2012). Carbonylation inhibits HDAC and HAT enzymatic activity, and is thereby thought to mediate the genome-wide changes in histone acetylation patterns typical of the inflammatory responses. Chromatin modification represents, also in filamentous fungi, a rapid and efficient way to switch gene expression on/off (Strauss and Reyes-Dominguez, 2011), and this control level applies to toxin biosynthetic genes, too (Li et al., 2009). Previous observations of oxylipin increases in plant and fungal cells during host-pathogen interactions led us to hypothesize that in a cascade effect, this would affect histone acetylation, thus modulating the global chromatin landscape and contributing to reprogramming the plant and fungal transcriptome. This, in turn, could affect also the expression of genes for secondary metabolite synthesis (mycotoxins included), and trigger fungal toxigenicity. Histone H4 proteins at the promoter of *FUM1* (P*FUM1*) are hyper-acetylated under FB-inducing conditions (Visentin et al., 2012). Our results indicate that oxylipins may control the transcription of *FUM* genes *via* local histone hyper-acetylation. Whether this happens through direct carbonylation and modulation of histone-modifying enzymes is still to be proven.

This study reports the *LDS1* gene duplication of our WT compared to the reference strain pinpointing the importance of oxylipin-related genes in these pathogenic fungi. It may be worth emphasizing here that gene duplication is thought to have a crucial role in evolution (Ohno, 1970). Gene duplication may result as a form of adaption when ecological stress occurs, leading to either dosage benefits or neo-functionalization of duplicated copies (Chang and Duda, 2012). In a recently proven, complementary theory, diversification of gene function predates duplication and stabilizes it. Subsequent specialization of the paralogs would allow an efficiency increase that outweighs fitness costs directly linked to gene duplication itself (Näsvall et al., 2012). In this perspective, it may be interesting to assess the transcription pattern of the only *LDS1* gene of the reference WT strain (7600) compared to *LDS1a* and *b* in our WT strain.

Experiments, aimed to reproduce the *in vivo* interaction among oxylipins and their potential target, may demonstrate how the lipid-derived signal can shape expression of the fungal genome, opening new perspectives in the understanding of how exogenous cues are perceived, transduced into reactive molecules and used for adapting the fungus to an ever-changing environment.

## Author contributions

Valeria Scala conceived of the study, participated in its design and coordination and helped to draft the manuscript. She performed the *in vitro* assays, generated the mutants, characterized them, and performed all the RT-qPCR assays. Paola Giorni performed the sexual reproduction and the pathogenicity assays. Martina Cirlini performed the fumonisin analysis. Matteo Ludovici performed the oxylipin LC-MS/MS analyses. Ivan Visentin performed the ChiP assay. Francesca Cardinale participated in the design of the study and coordination and helped to draft the manuscript. Anna A. Fabbri conceived of the study, participated in its design and coordination and helped to draft the manuscript. Corrado Fanelli conceived of the study, participated in its design and coordination and helped to draft the manuscript. Massimo Reverberi conceived of the study, participated in its design and coordination and helped to draft the manuscript. Paola Battilani conceived of the study, and participated in its design and coordination and helped to draft the manuscript. Gianni Galaverna participated to the design of the study. Chiara Dall'Asta conceived of the study, participated in its design and coordination and helped to draft the manuscript. All authors read and approved the final manuscript.

## Funding

The present study was funded by the Ministry of Research and Education through the project FIRB2008 “Futuro in Ricerca,” grant N. FIRB-RBFR08JKHI.

### Conflict of interest statement

The authors declare that the research was conducted in the absence of any commercial or financial relationships that could be construed as a potential conflict of interest.

## Supplementary material

Supplementary Image 1 **Characterization of the genomic organization of Fv*LDS1* in WT *F. verticillioides* and putative Fv*lds1*-deleted D strain (ΔFv*lds1*D)**. Southern blot hybridization of *Eco*RI-restricted genomic DNA was carried out using PCR digoxigenin (DIG)-labeled fragments of *LDS1* and *Hph* as molecular probes.

Supplementary Image 2 **(A)** Duplicated region inferred with a depth of coverage approach. The X axis shows the Supercontig coordinates and the Y axis the depth of coverage. The black line shows for each 100 bp bin the depth of coverage and the green one the normalized coverage value. The blue lines highlight the *LDS1* locus. **(B)** Non-duplicated region inferred with a depth-of-coverage approach. The X axis shows the Supercontig coordinates and the Y axis the depth of coverage. The black line shows for each 100 bp bin the depth of coverage and the green one the normalized coverage value. The blue lines highlight the *LDS1* locus.

Supplementary Image 3 **Fv*lds1* complementation**. Complemented strains were generated by transformation of the ΔFv*lds1*D mutant with the WT *LDS1b* allele using the geneticin resistance gene, *GenR*, as a selectable marker. The figure shows the results of PCR-screening of five putative complementation strains using primers designed on the *GenR* box.

Supplementary Image 4 **Sexual fertility assays**. Images of sexual crossings and summary of the presence of perithecia (reported also as mean percentage of 5 repetitions) after 8 weeks of incubation between *F. verticillioides* WT and ΔFv*lds1*D with the opposite MAT-type reference strain of *F. verticillioides* (ITEM 15574). Agarose gel of PCR products amplified from DNA isolated from *F. verticillioides* ITEM 10027 (WT) and its mutant ΔFv*lds1*D. The *F. verticillioides*-specific fusALPHA primers (MAT-1) were capable of amplifying; not so fusHMG primers (MAT-2).

Supplementary Data Sheet 1 **Oxylipin production *in vitro***. MRM analysis of 17 different oxylipins in *F. verticillioides* WT, ΔFv*lds1*D and COM strains at different days after inoculation (2–15 DAI) under *in vitro* conditions (see Materials and Methods section for growing conditions). Results are the mean (± SE) of six replications from two independent experiments and are expressed as μM.

Supplementary Data Sheet 2 **Oxylipin production *in vivo***. MRM analysis of 15 different oxylipins in maize infected with *F. verticillioides* WT and ΔFv*lds1*D strains or non-infected (mock) at different days after inoculation (2–15 DAI) under *in vivo* conditions (see Materials and Methods section for inoculation conditions into maize cobs). Results are the mean (± SE) of six replications from two independent experiments and are expressed as μM.

Supplementary Table 1 **Primers used in this study**.

## Abbreviations

CDYM: Basal medium with the addition of 0.2% (w/v) cracked maize
ChIP: chromatin immunoprecipitation
CNV: copy number variation
DAI: days after inoculation
diHODE: dihydroxyoctadecenoic acid
FAs: Fatty acids
FB_1_: fumonisin B_1_
HATs: histone acetyl transferases
HDACs: histone deacetylases
HPODE: hydroperoxyoctadecenoic acid
LDS1: Linoleate Diol Synthase 1
LOX: lipoxygenases
MRM: multiple reaction monitoring
PR: pathogenesis-related
P*FUM1*: promoter of *FUM1*
RD: read depth
RT-qPCR: reverse transcription quantitative PCR
SVs: structural variations
FB_tot_: total fumonisins.
