# Supplementary figures and images for: LDS1-produced oxylipins are negative regulators of growth, conidiation and fumonisin synthesis in the fungal maize pathogen Fusarium verticillioides
Source: Front Microbiol. 2014 Dec 11;5:669. doi: 10.3389/fmicb.2014.00669 (PMC4263177; doi:10.3389/fmicb.2014.00669)

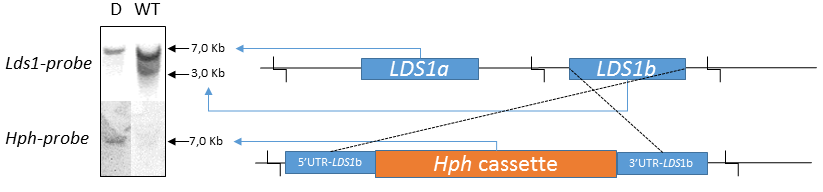

Supplement: Supplementary Image 1 — Characterization of the genomic organization of FvLDS1 in WT F. verticillioides and putative Fvlds1-deleted D strain (ΔFvlds1D). Southern blot hybridization of EcoRI-restricted genomic DNA was carried out using PCR digoxigenin (DIG)-labeled fragments of LDS1 and Hph as molecular probes. [file Image1.TIF]

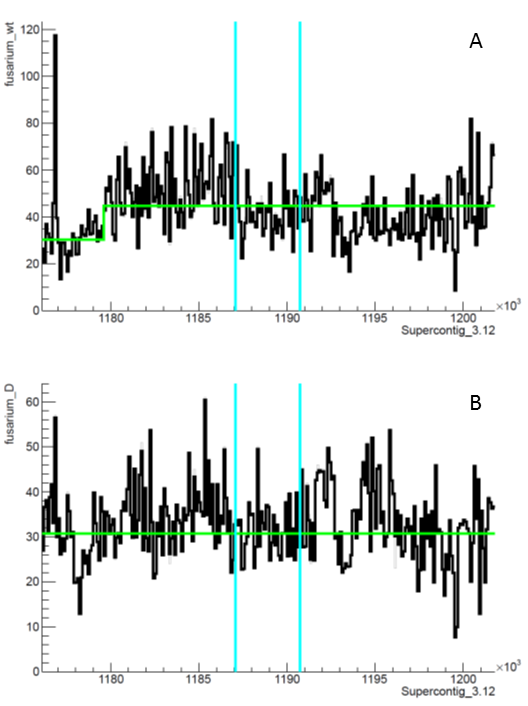

Supplement: Supplementary Image 2 — (A) Duplicated region inferred with a depth of coverage approach. The X axis shows the Supercontig coordinates and the Y axis the depth of coverage. The black line shows for each 100 bp bin the depth of coverage and the green one the normalized coverage value. The blue lines highlight the LDS1 locus. (B) Non-duplicated region inferred with a depth-of-coverage approach. The X axis shows the Supercontig coordinates and the Y axis the depth of coverage. The black line shows for each 100 bp bin the depth of coverage and the green one the normalized coverage value. The blue lines highlight the LDS1 locus. [file Image2.TIF]

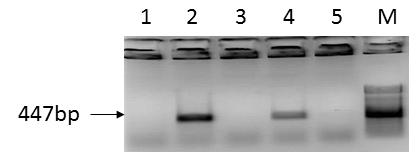

Supplement: Supplementary Image 3 — Fvlds1 complementation. Complemented strains were generated by transformation of the ΔFvlds1D mutant with the WT LDS1b allele using the geneticin resistance gene, GenR, as a selectable marker. The figure shows the results of PCR-screening of five putative complementation strains using primers designed on the GenR box. [file Image3.TIF]

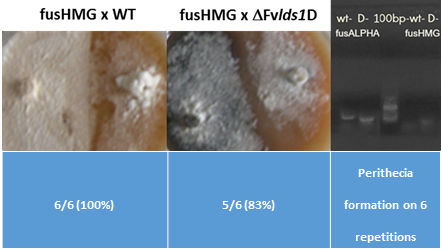

Supplement: Supplementary Image 4 — Sexual fertility assays. Images of sexual crossings and summary of the presence of perithecia (reported also as mean percentage of 5 repetitions) after 8 weeks of incubation between F. verticillioides WT and ΔFvlds1D with the opposite MAT-type reference strain of F. verticillioides (ITEM 15574). Agarose gel of PCR products amplified from DNA isolated from F. verticillioides ITEM 10027 (WT) and its mutant ΔFvlds1D. The F. verticillioides-specific fusALPHA primers (MAT-1) were capable of amplifying; not so fusHMG primers (MAT-2). [file Image4.TIF]

**LDS-related oxylipins**


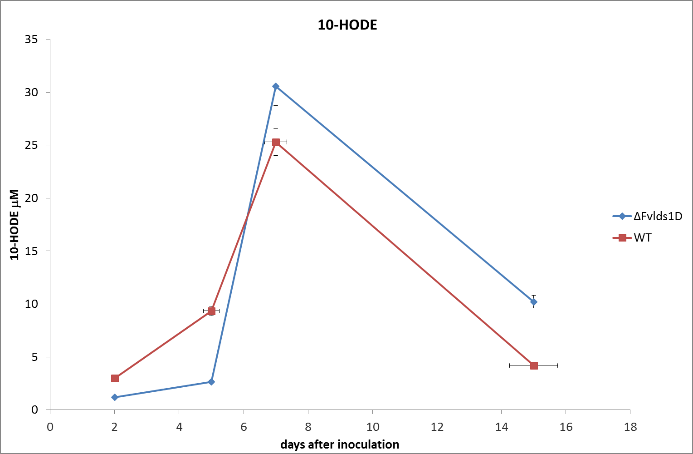

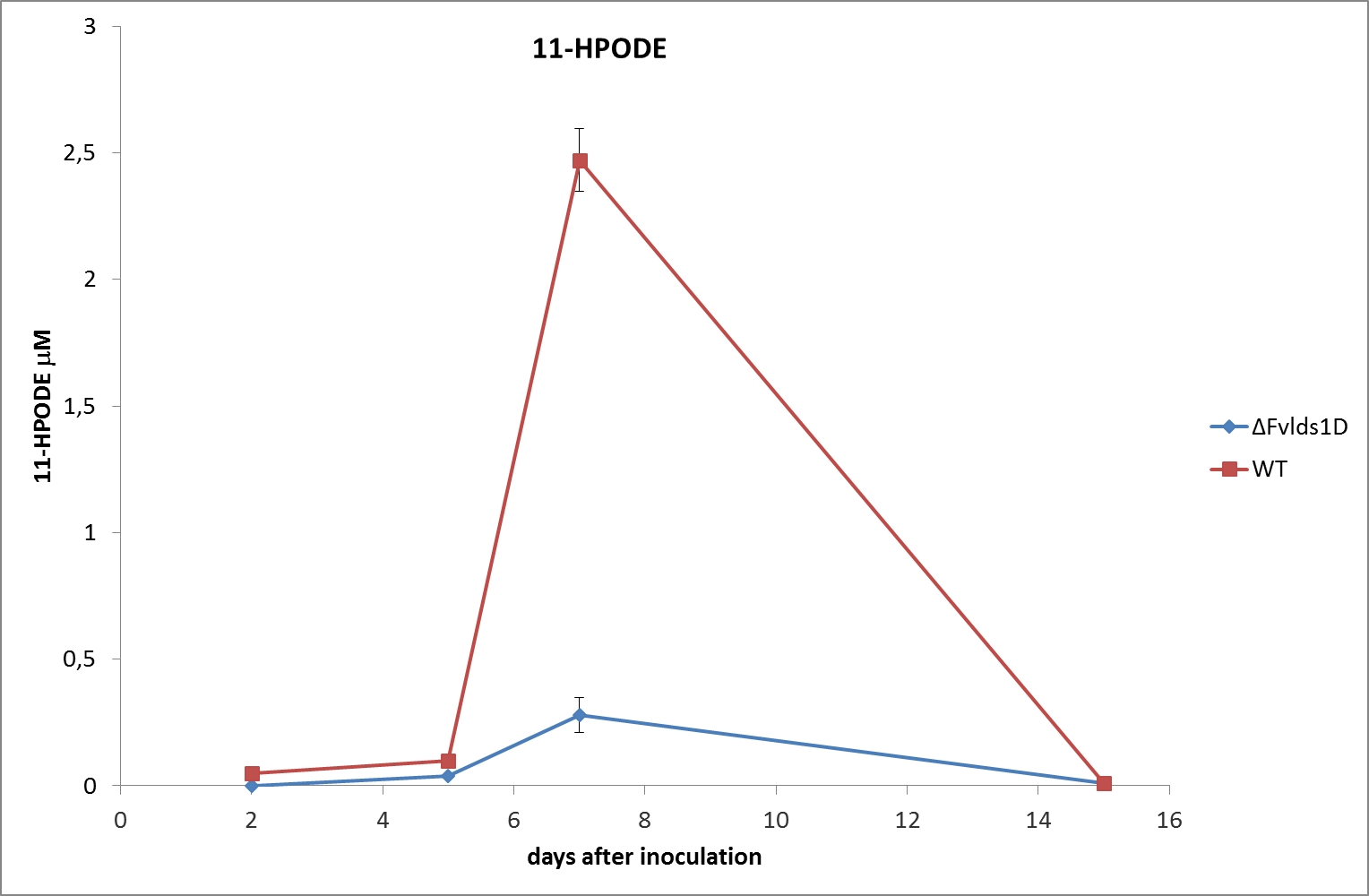


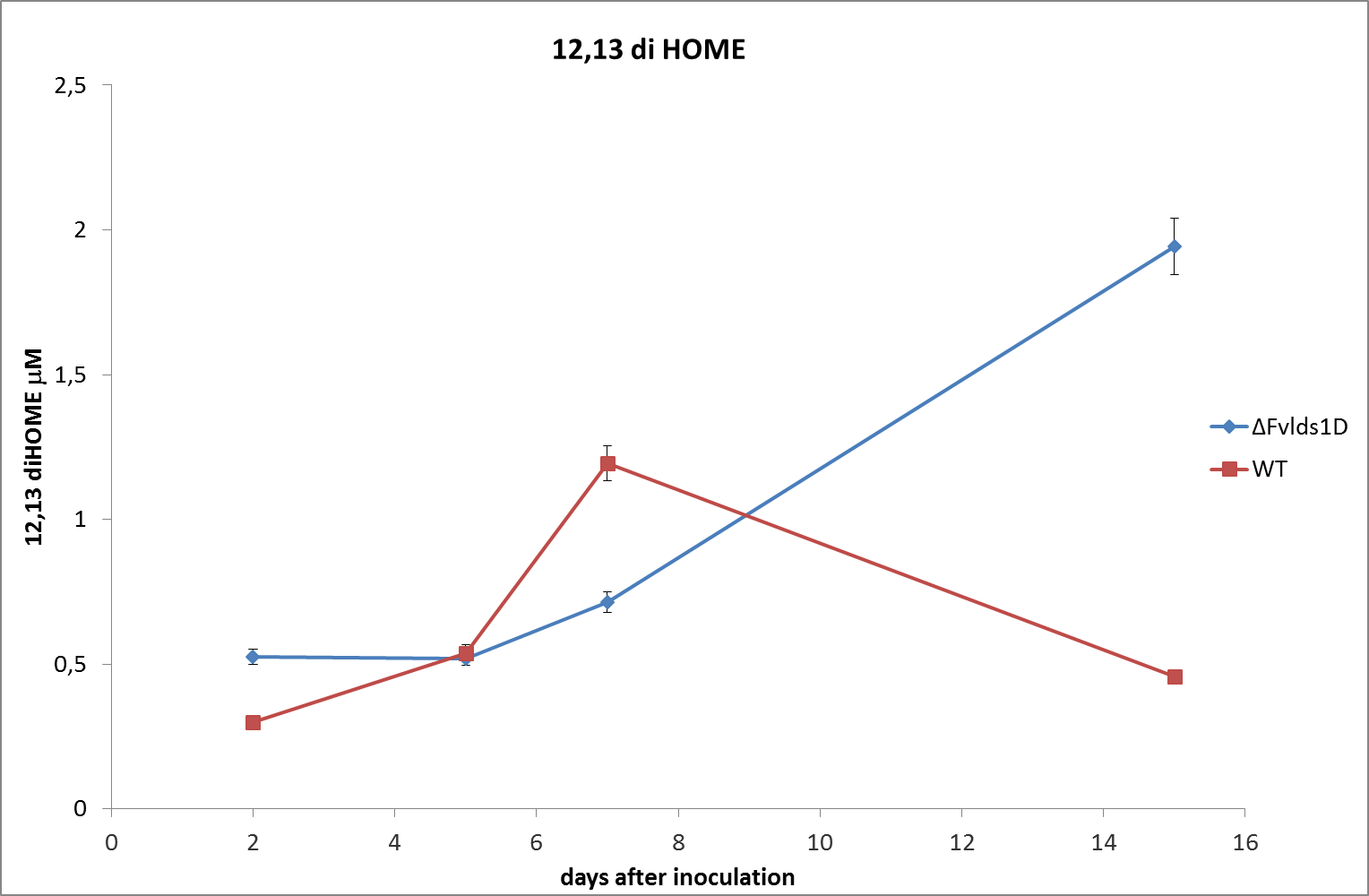

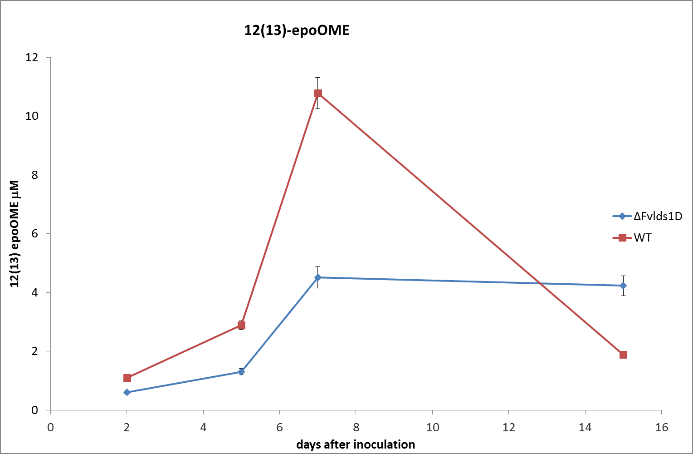


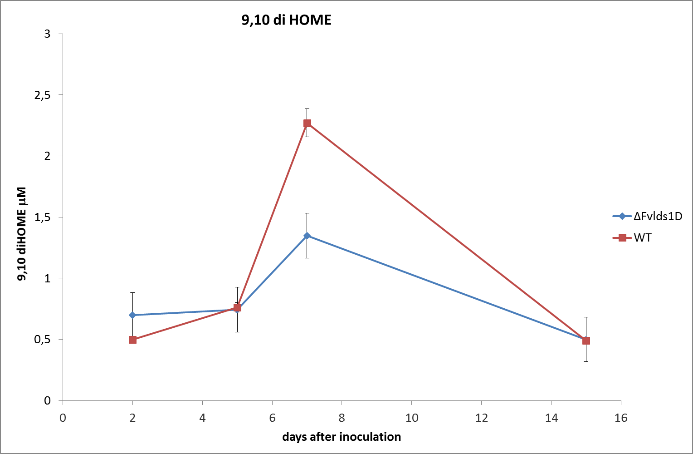

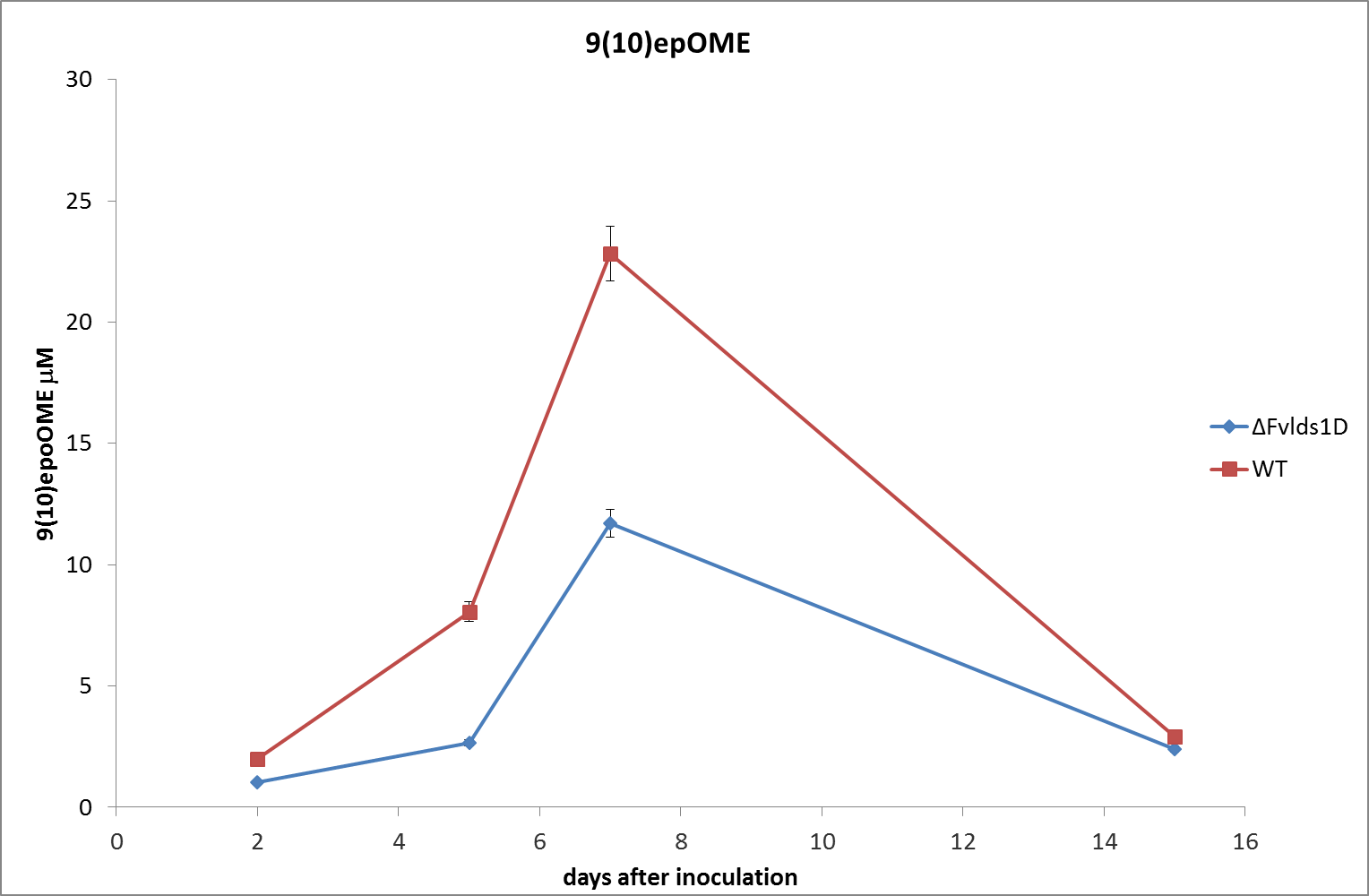


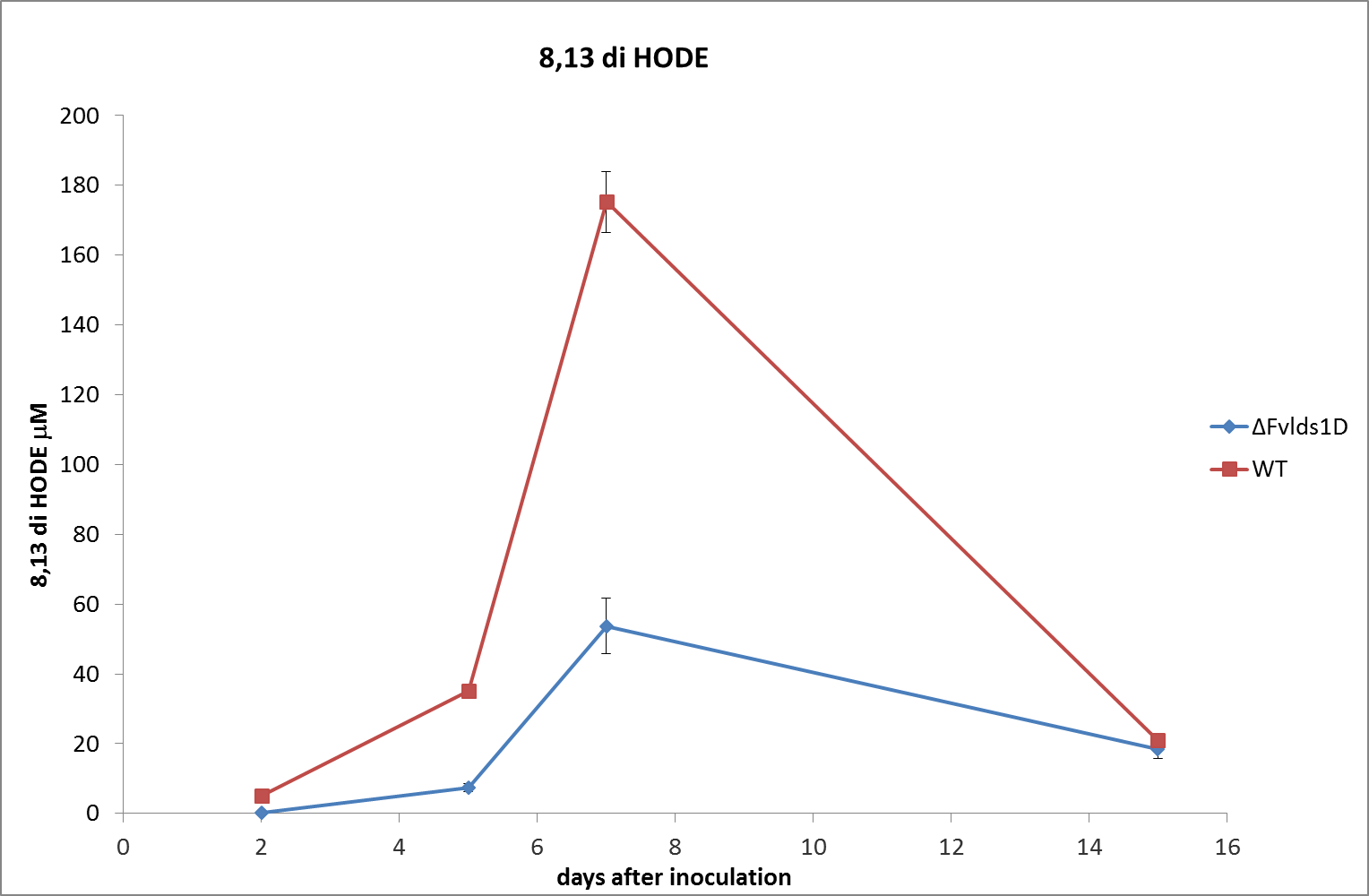

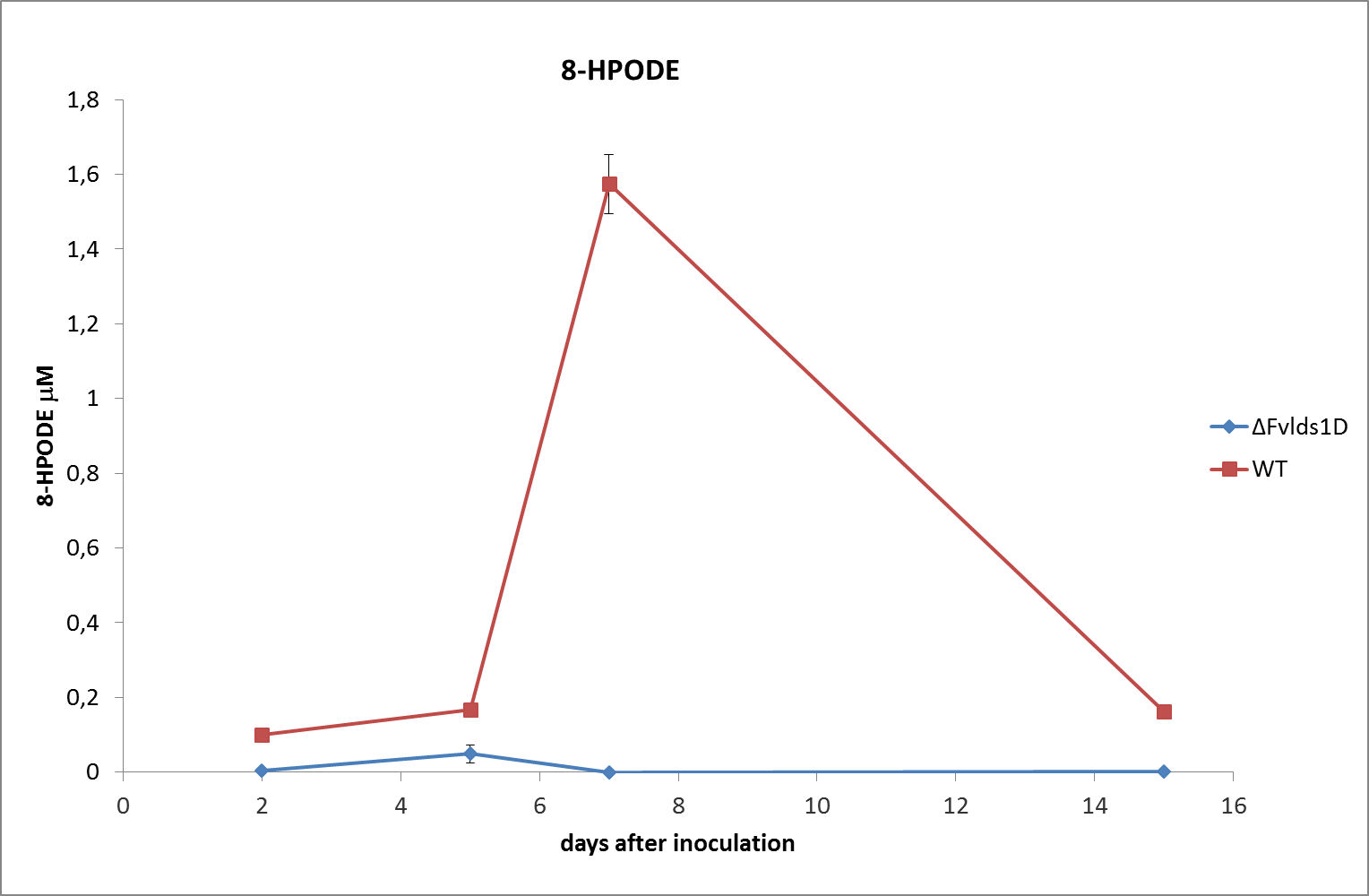


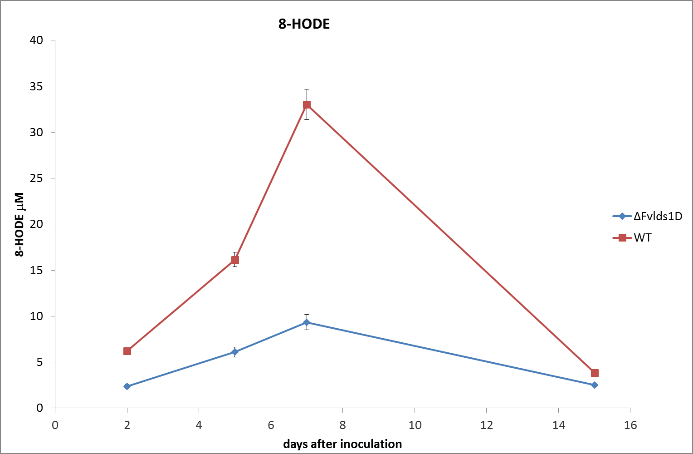


**LOX-related oxylipins**


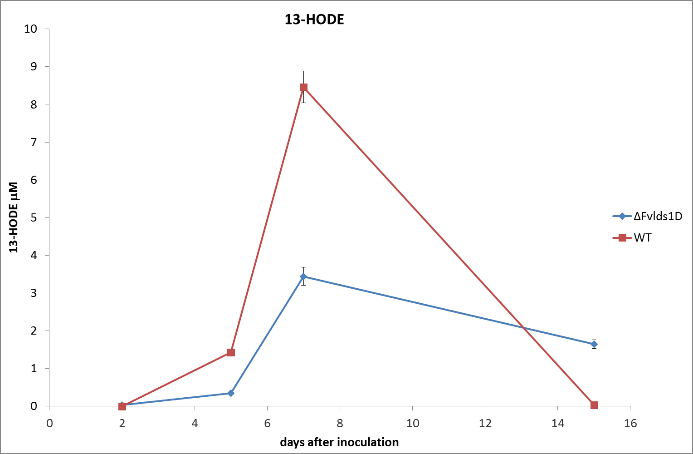

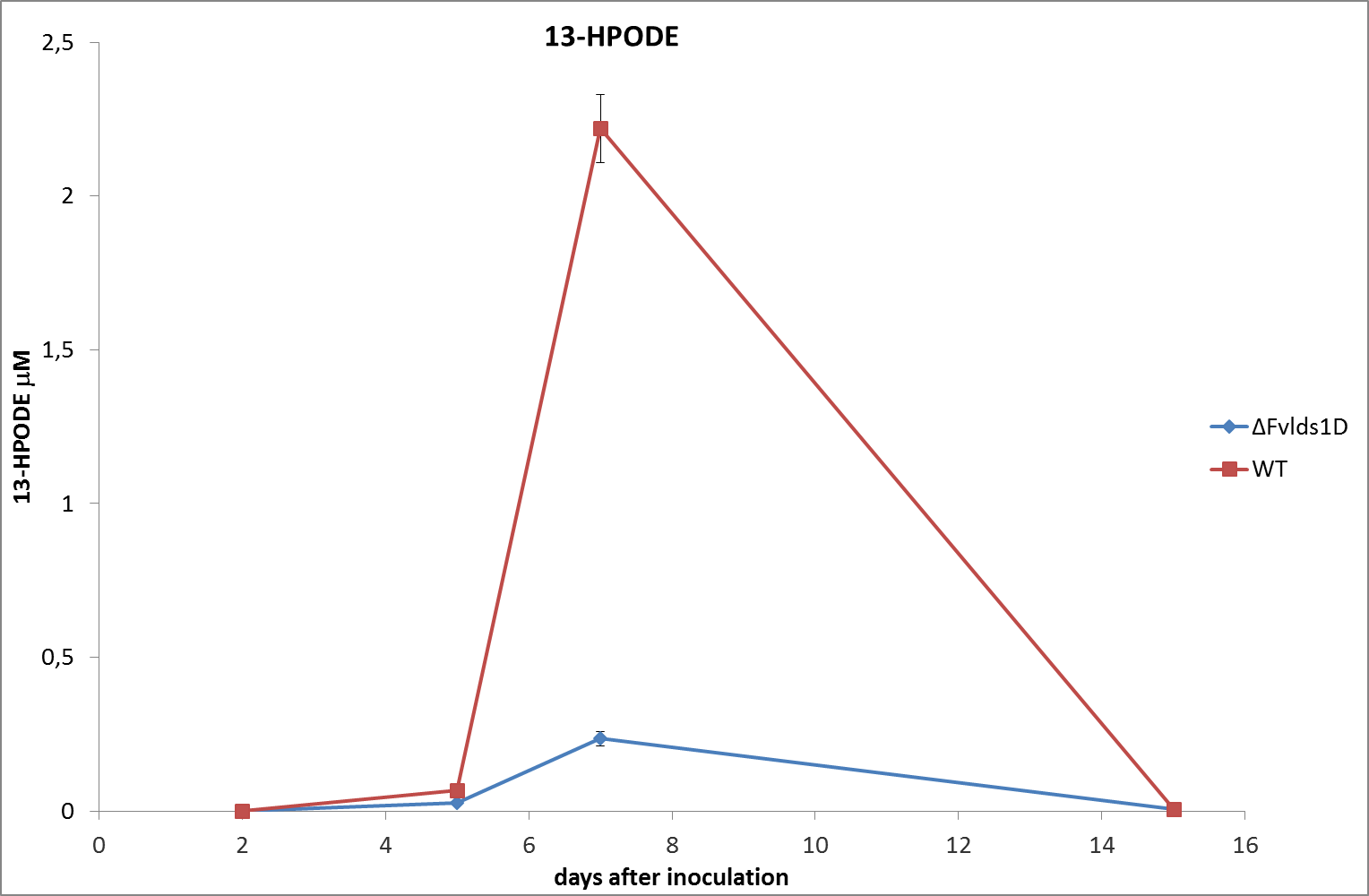

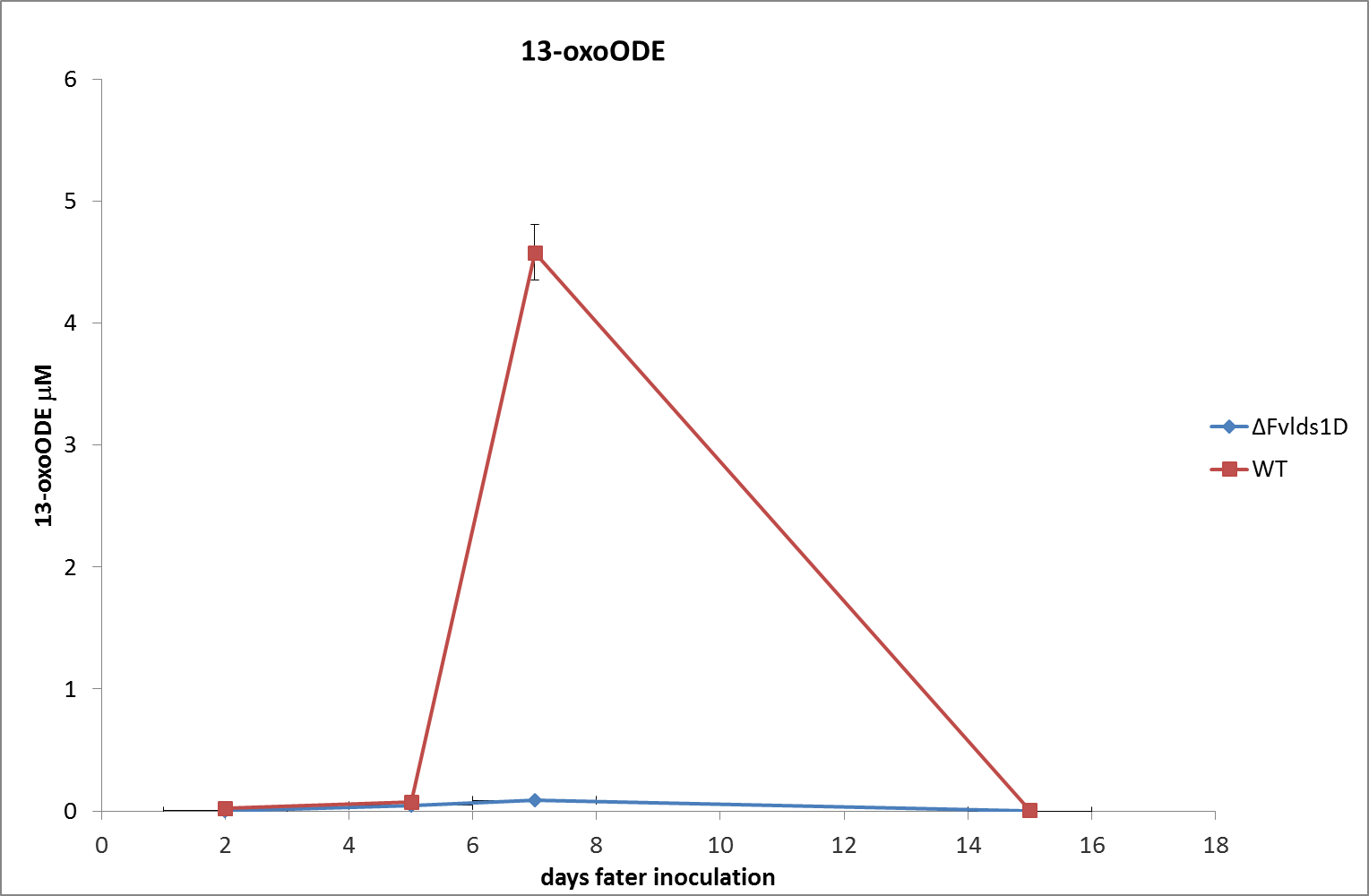

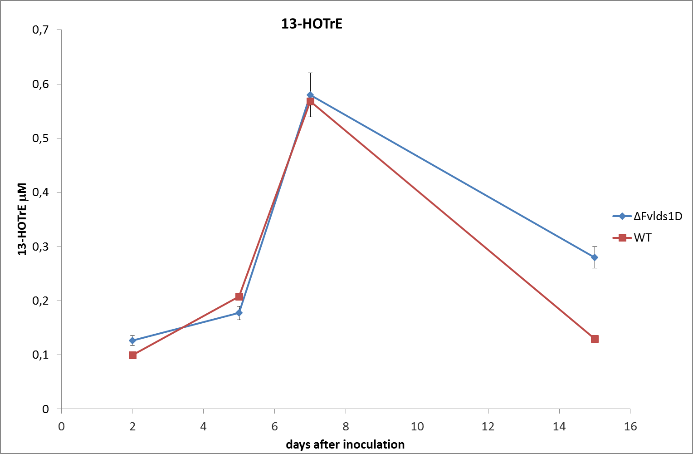

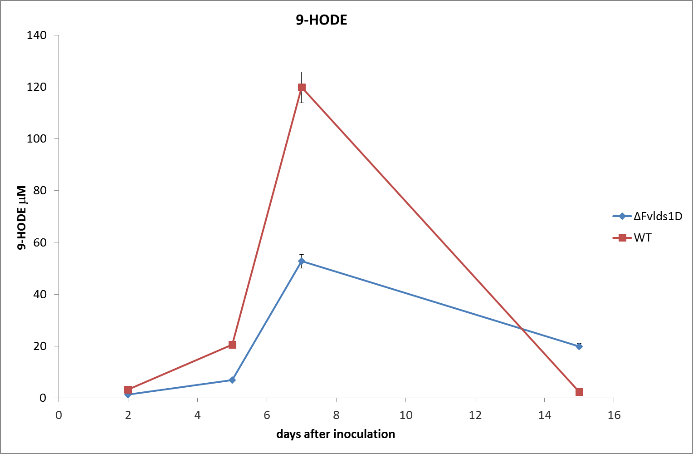

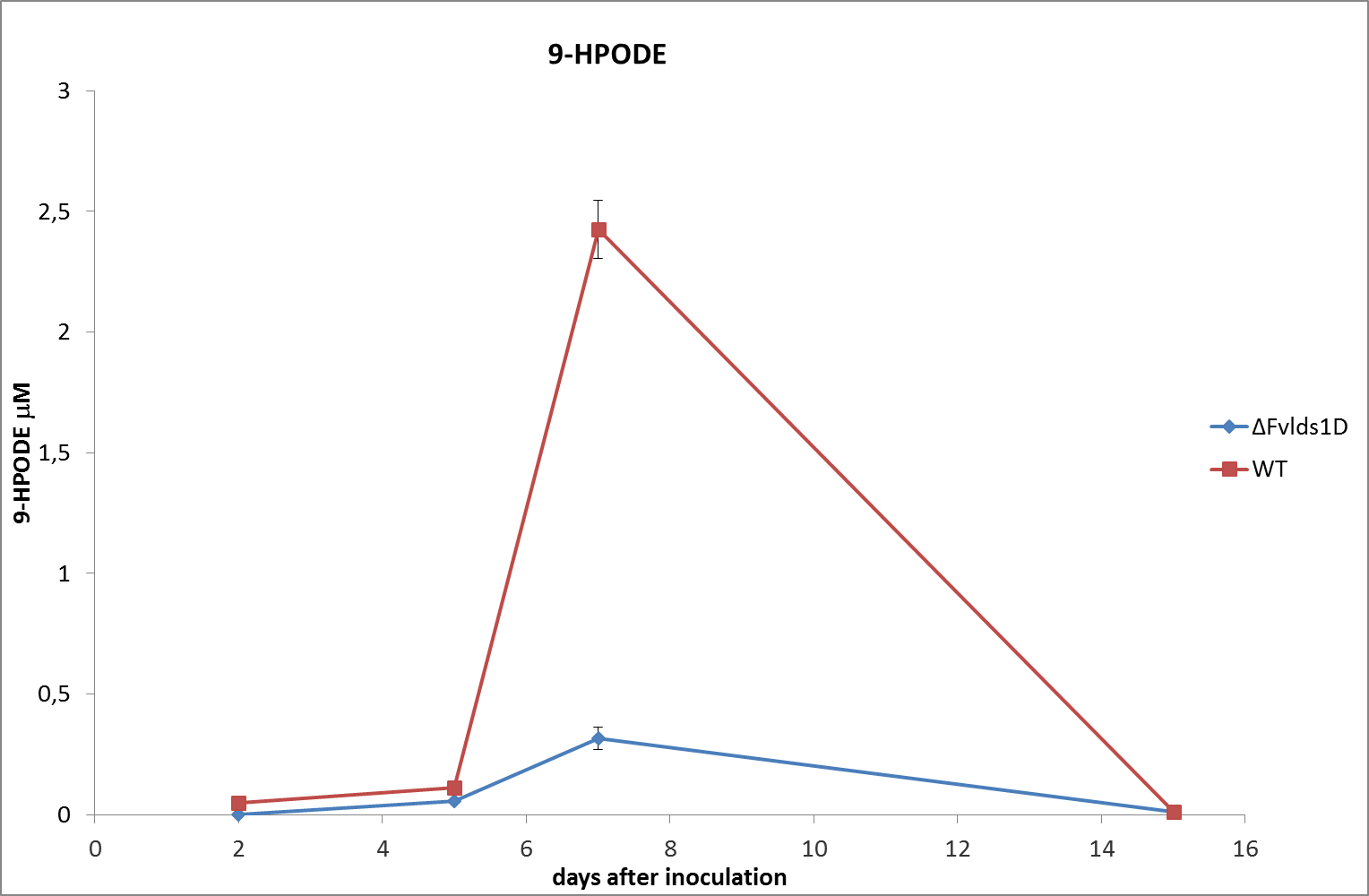

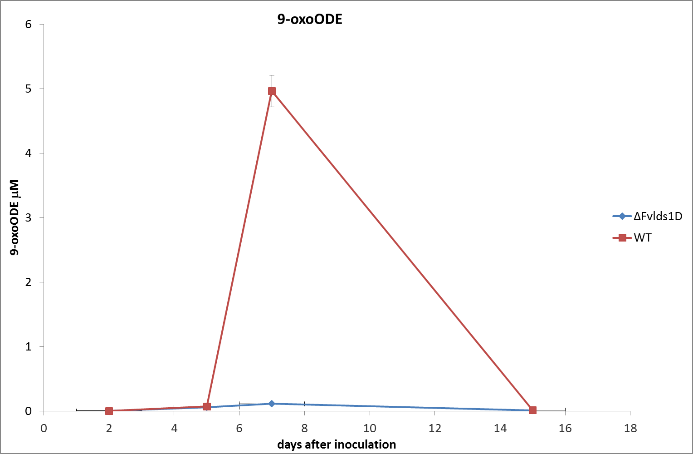

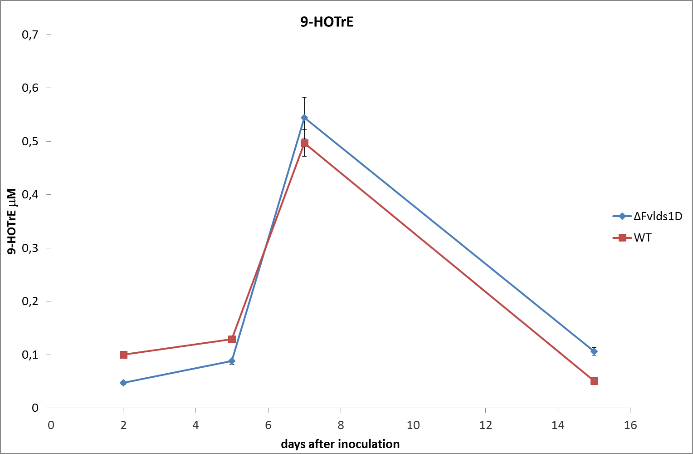

Supplement: Supplementary Data Sheet 1 — Oxylipin production in vitro. MRM analysis of 17 different oxylipins in F. verticillioides WT, ΔFvlds1D and COM strains at different days after inoculation (2–15 DAI) under in vitro conditions (see Materials and Methods section for growing conditions). Results are the mean (± SE) of six replications from two independent experiments and are expressed as μM. [file DataSheet1.DOCX]

**LDS-derived oxylipins**


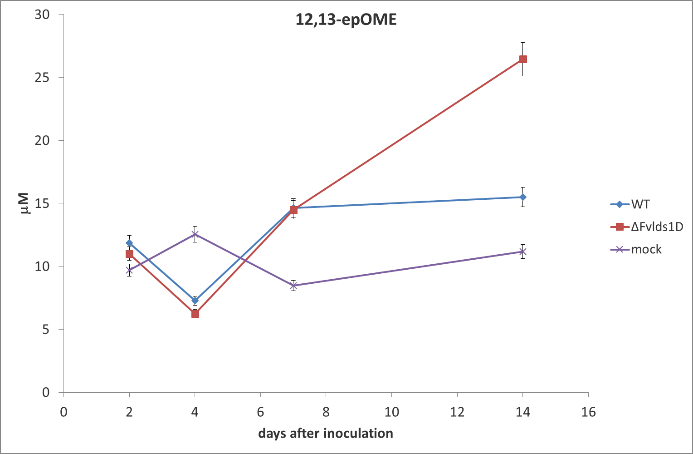

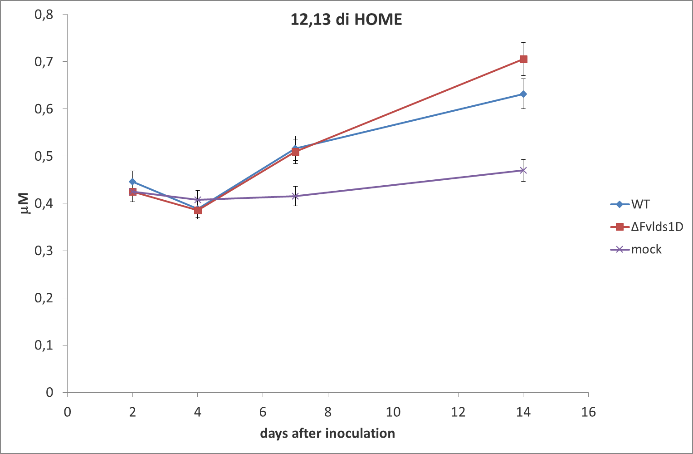


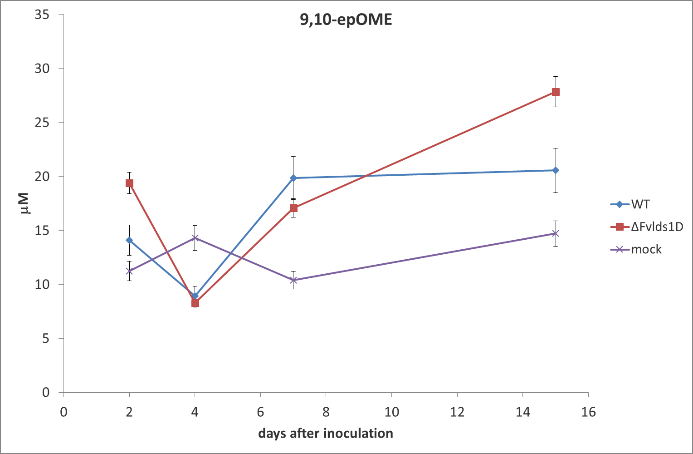

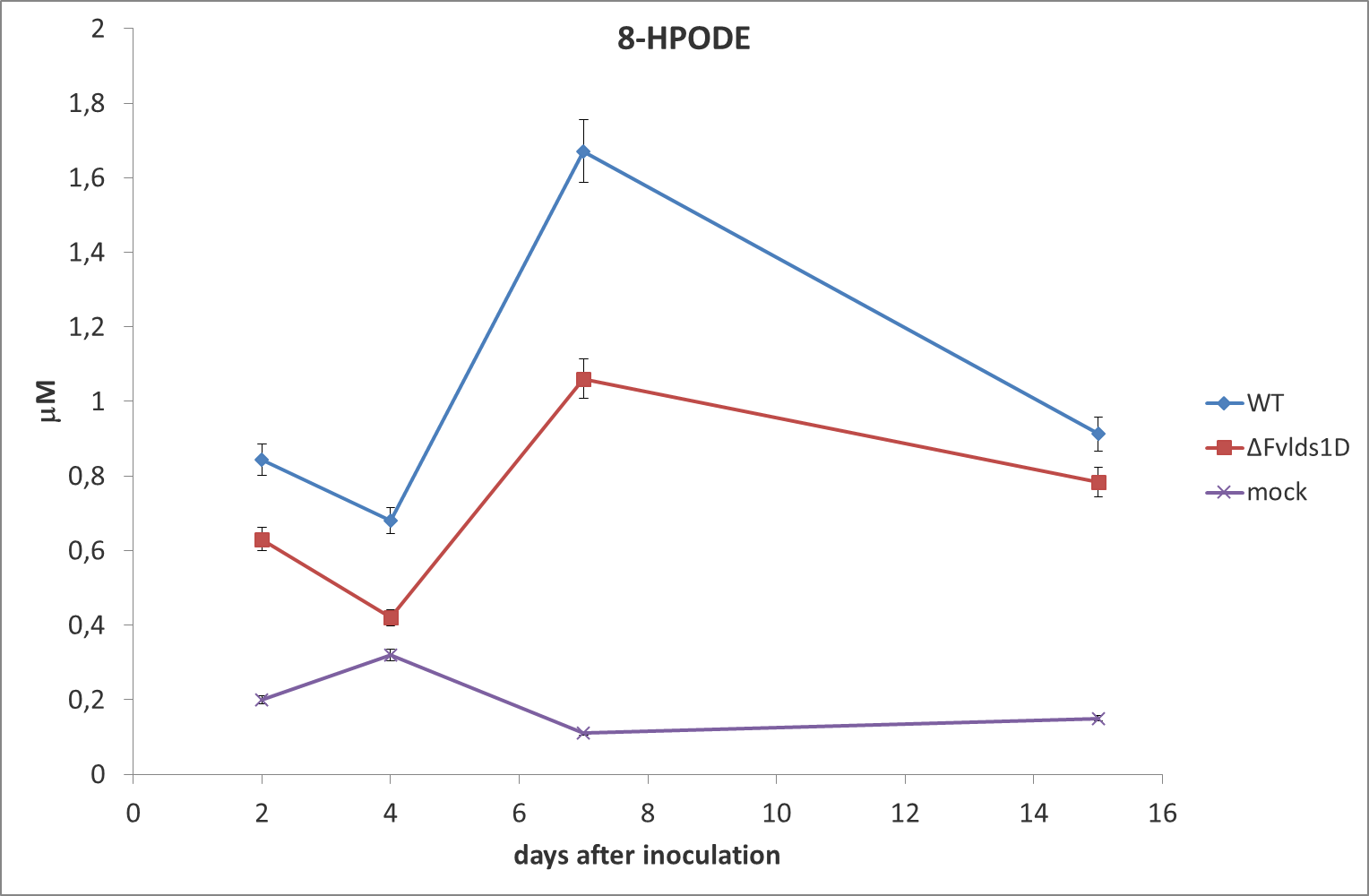

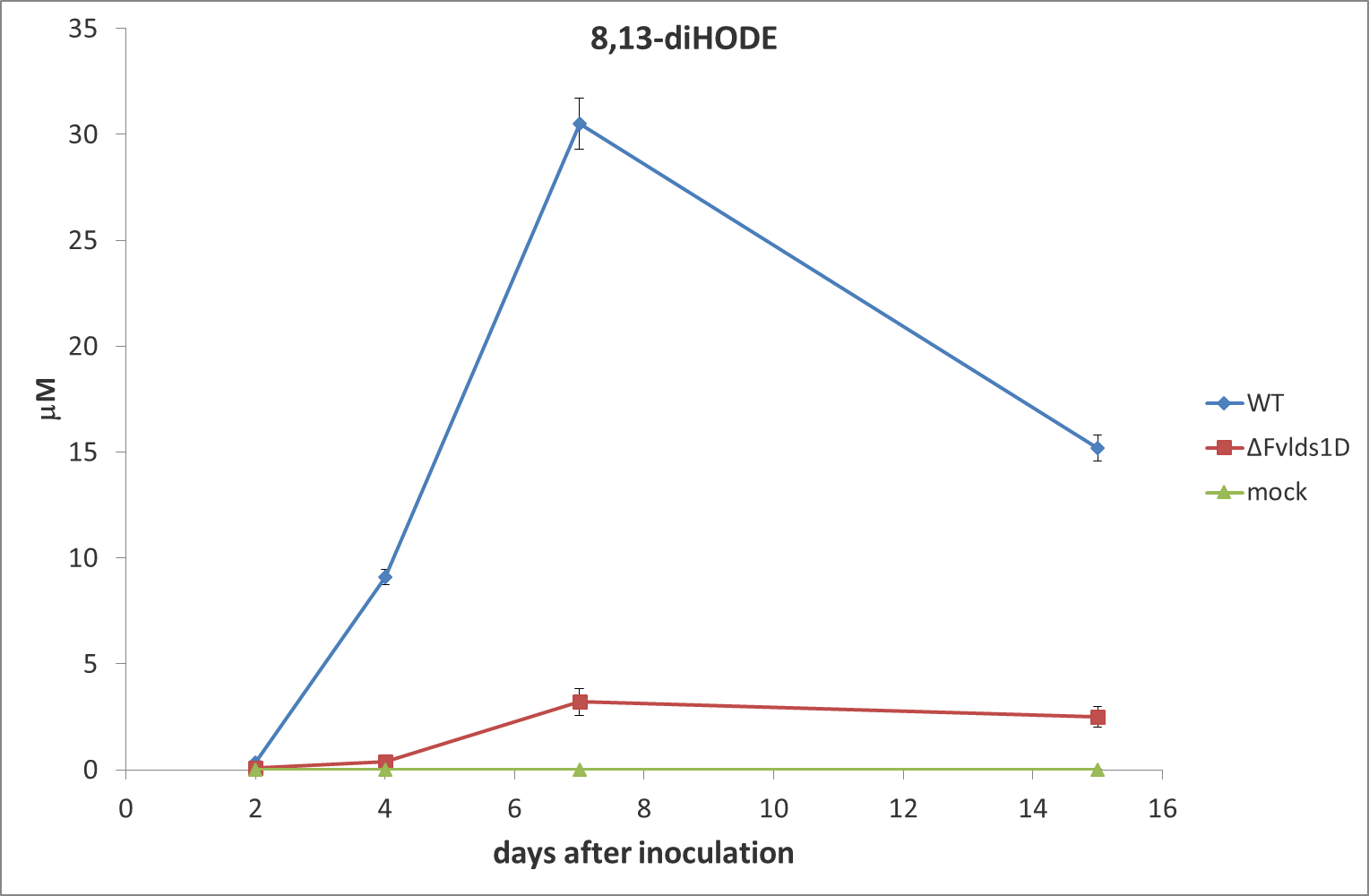

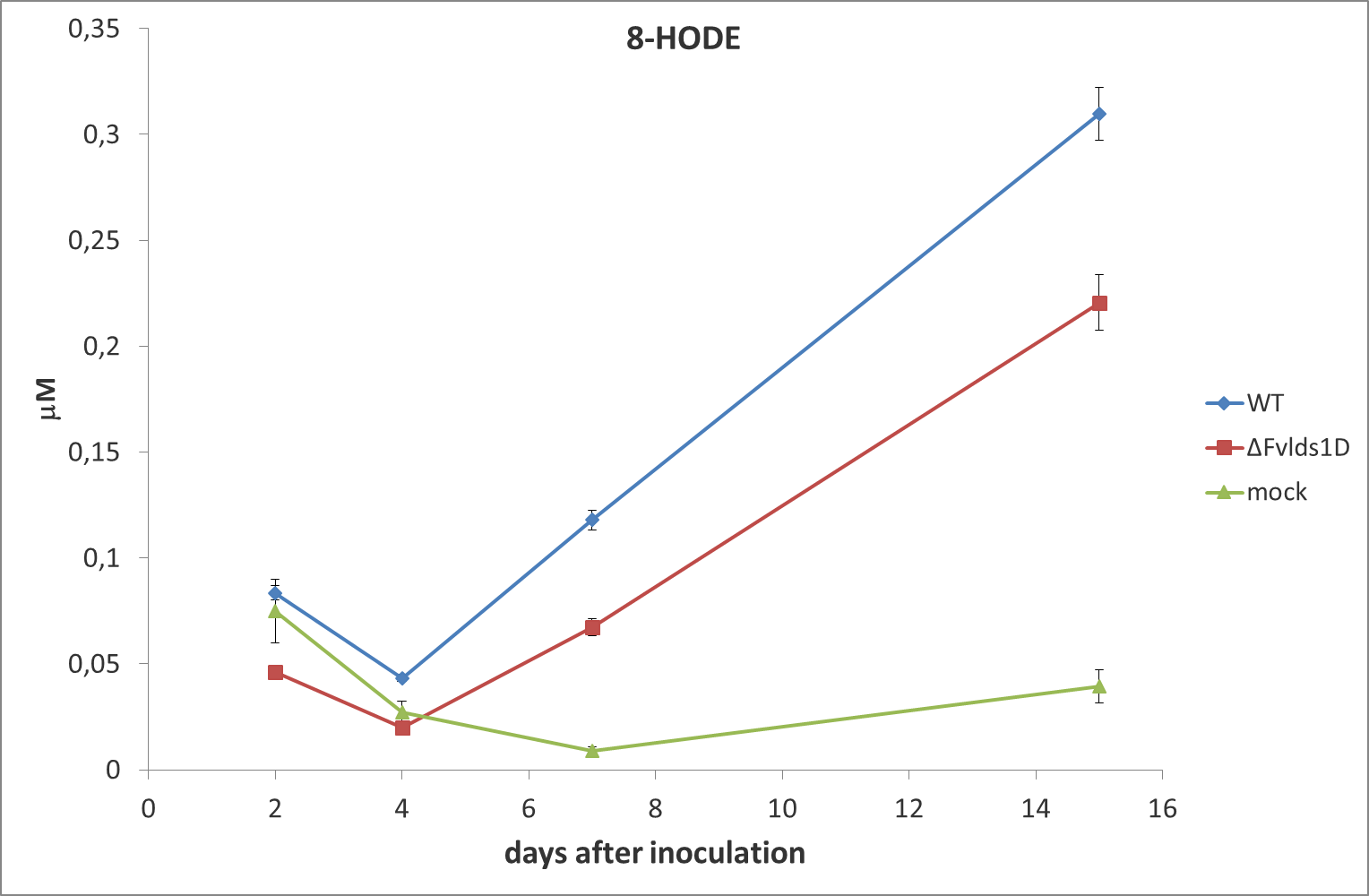

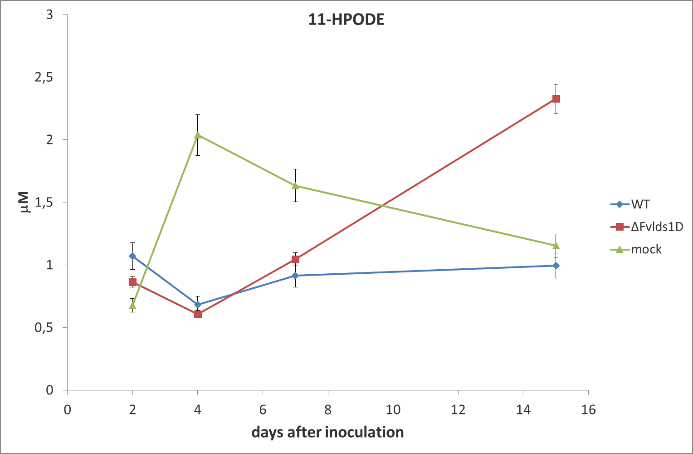


**LOX-derived oxylipins**


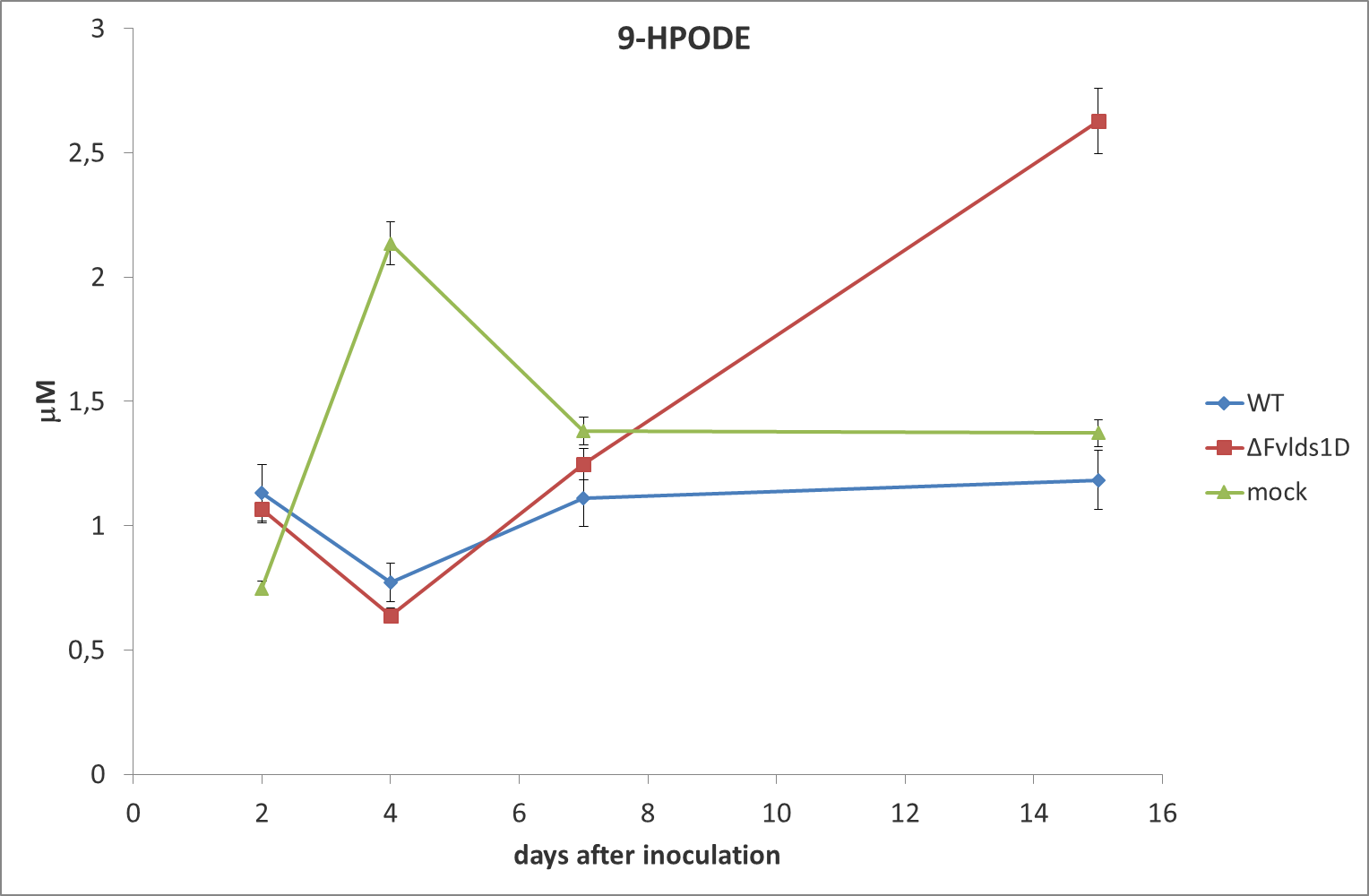

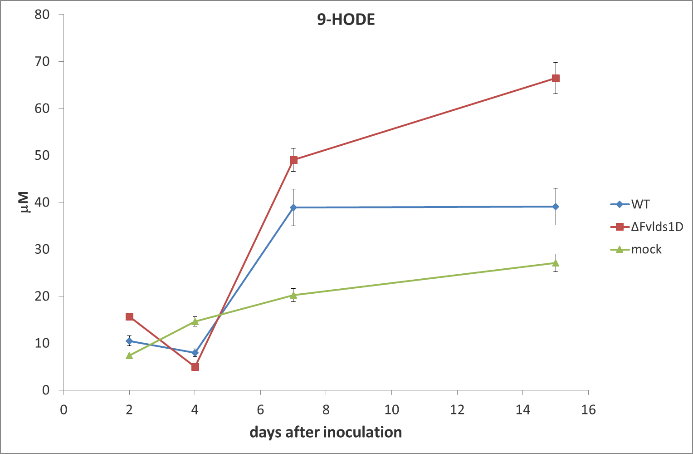

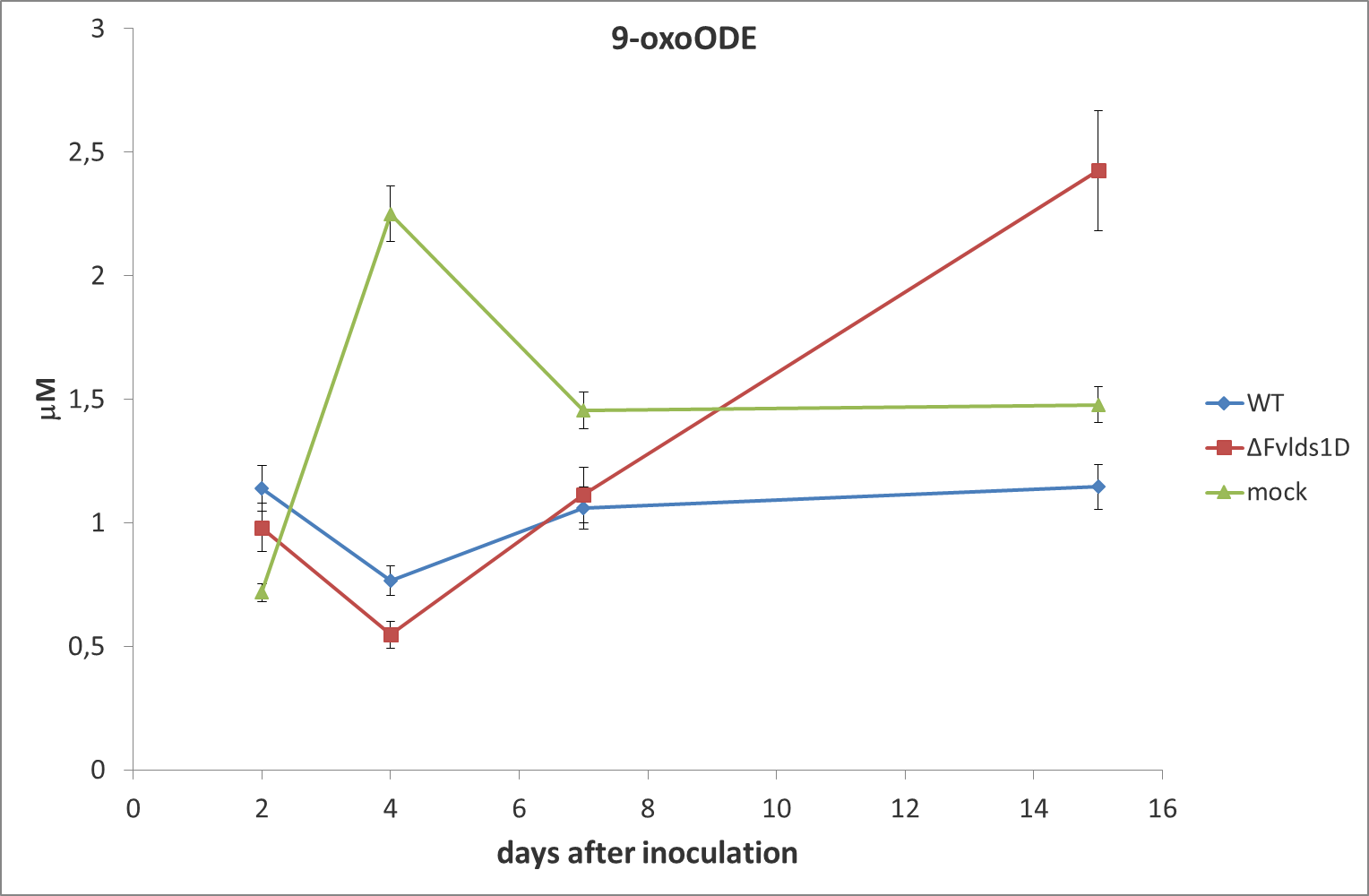

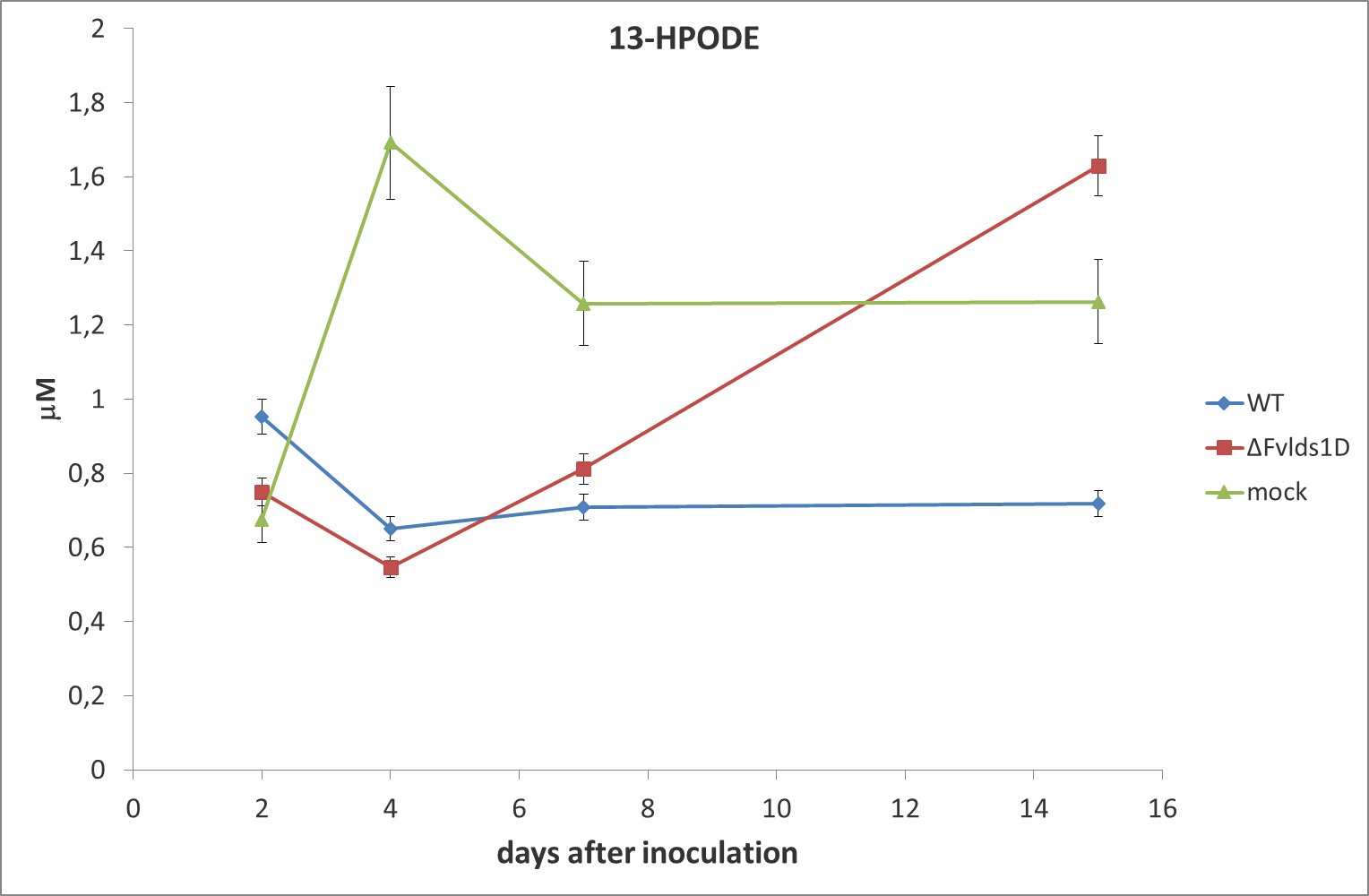


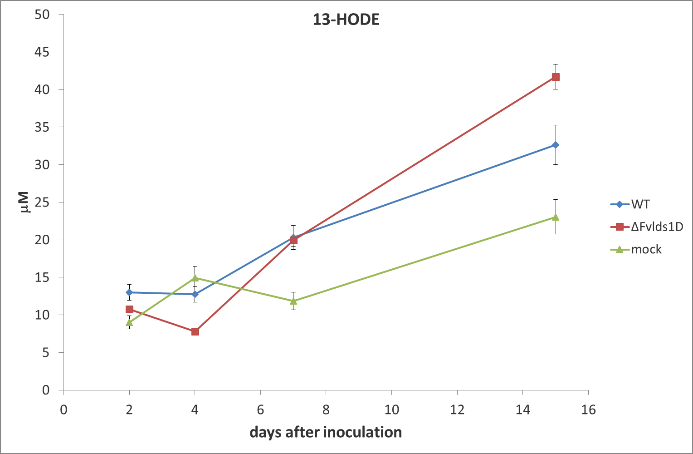

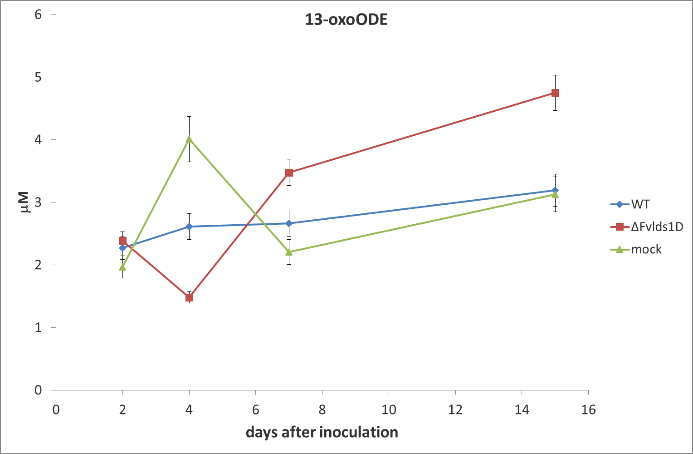


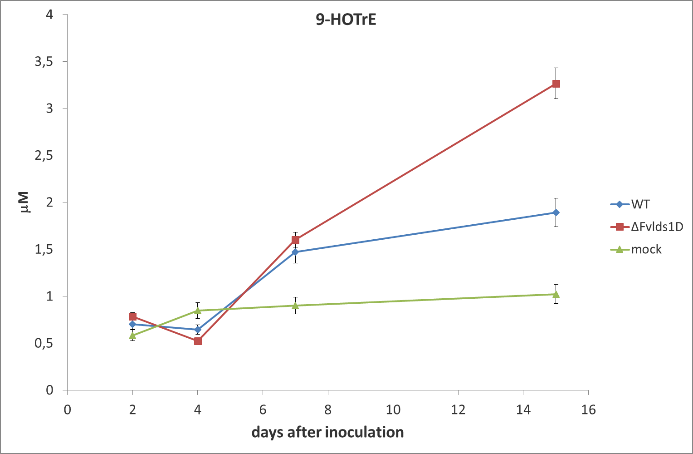

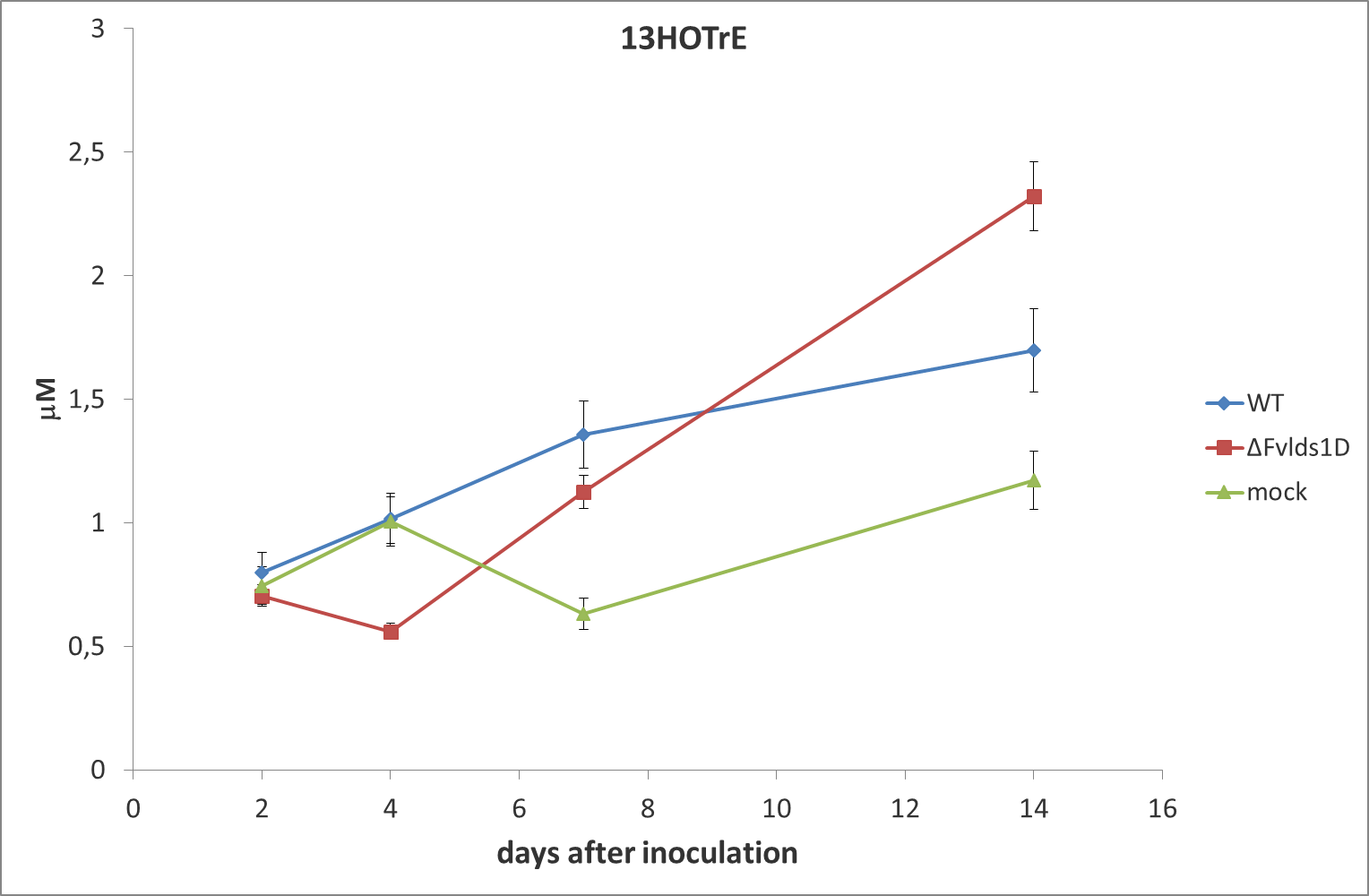

Supplement: Supplementary Data Sheet 2 — Oxylipin production in vivo. MRM analysis of 15 different oxylipins in maize infected with F. verticillioides WT and ΔFvlds1D strains or non-infected (mock) at different days after inoculation (2–15 DAI) under in vivo conditions (see Materials and Methods section for inoculation conditions into maize cobs). Results are the mean (± SE) of six replications from two independent experiments and are expressed as μM. [file DataSheet2.DOCX]
