# Supplementary material for: LDS1-produced oxylipins are negative regulators of growth, conidiation and fumonisin synthesis in the fungal maize pathogen Fusarium verticillioides
Source: Front Microbiol. 2014 Dec 11;5:669. doi: 10.3389/fmicb.2014.00669 (PMC4263177; doi:10.3389/fmicb.2014.00669)
Supplement: Supplementary Table 1 — Primers used in this study. [file Table1.DOCX]

**Supplementary Table 1. Primers used in this study.**

| **Primer name** | **Primer sequence** |
| --- | --- |
| Lds_for_PciI | ACATGTGACTGTCAAGTTGCGTTTCG |
| Lds_for_StuI | ACGAATGCATCGCCAAAG |
| Lds_for_NaeI | GCCGGCGTCCGCAACGAGTTTCTCAG |
| Lds_for_SbfI | CCTGCAGGCAACCTGTCTCGCCCATATC |
| panlds1F | GACTGAGGAATCCGCTCTTG |
| panldsR | TCATTGTCTCAGCTCGCATC |
| Lds1_for | GCGATGGAGTTGAAGGGGAA |
| Lds1_rev | TCCTCGTGTTGCGATTGACA |
| nptII_for | AGCCGGTATAAAGGGACCAC |
| nptII_rev | GATGTTGCTGTCTCCCAGGT |
| *Fum1_*for | GAGCCGAGTCAGCAAGGATT |
| *Fum1_*rev | AGGGTTCGTGAGCCAAGGA |
| PFUM1_for | CTCTATGCCAGCCCTGACTC |
| PFUM1_rev | GGGACAGTAACGTCCGAAAA |
| ZmPR4_for | CTCGATGCTTCGCCGTATC |
| ZmPR4_rev | GGCAACCGATCGATGTAG |
| Zm actin for | TCCTGACACTGAAGTCCCGATTG |
| Zm actin rev | CGTTGTAGAAGGTGTGATGCCAGTT |
